# Liquid Phase Separation of NEMO Induced by Polyubiquitin Chains Activates NF-κB

**DOI:** 10.1016/j.molcel.2022.03.037

**Published:** 2022-04-26

**Authors:** Mingjian Du, Chee-Kwee Ea, Yan Fang, Zhijian J. Chen

**Affiliations:** 1 Department of Molecular Biology, University of Texas Southwestern Medical Center, Dallas, TX 75390-9148, USA; 2 Center for Inflammation Research, University of Texas Southwestern Medical Center, Dallas, TX 75390-9148, USA; 3 Howard Hughes Medical Institute, University of Texas Southwestern Medical Center, Dallas, TX 75390-9148, USA; 4 Lead Contact

## Abstract

The NF-κB essential modulator (NEMO) is a regulatory subunit of the IκB kinase (IKK) complex that phosphorylates the NF-κB inhibitors IκBs. NEMO mediates IKK activation by binding to polyubiquitin chains (polyUb). Here we show that Lys63(K63)-linked or linear polyUb binding to NEMO robustly induced the formation of liquid-like droplets in which IKK was activated. This liquid phase separation of NEMO was driven by multivalent interactions between NEMO and polyUb. Both the NEMO-ubiquitin-binding (NUB) domain and the zinc finger (ZF) domain of NEMO mediated binding to polyUb and contributed to NEMO phase separation and IKK activation in cells. Moreover, NEMO mutations associated with human immunodeficiency impaired its phase separation. These results demonstrate that polyUb activates IKK and NF-κB signaling by promoting the phase separation of NEMO.

## INTRODUCTION

NF-κB is a transcription factor that regulates diverse cellular processes, including immune and inflammatory responses (Sen and Baltimore, 1986a; b; Zhang et al., 2017). NF-κB, which is a dimer consisting of members of the Rel family of proteins including p50 and p65, is normally sequestered in the cytoplasm by an inhibitory protein of the IκB family. In response to stimulation by an agent such as interleukin-1β (IL-1β) or tumor necrosis factor α (TNFα), the IκB kinase complex (IKK), which consists of IKKα, IKKβ and NEMO (also known as IKKγ), is activated to phosphorylate IκBs. The phosphorylated IκB proteins are then polyubiquitinated and degraded by the proteasome, leading to translocation of NF-κB into the nucleus, where it activates the expression of many genes including those involved in immune and inflammatory responses.

IL-1 receptors bear an intracellular Toll/Interleukin-1 receptor (TIR) domain that when activated can bind the TIR domain of the adapter protein myeloid differentiation primary response 88 (MyD88), which recruits the IL-1 receptor-associated kinase 4 (IRAK4). IRAK4 then recruits and phosphorylates IRAK1 (and IRAK2). Phosphorylated IRAK1 dissociates from IRAK4 and activates the E3 ligase TRAF6 (Chen, 2012). TRAF6 functions together with the ubiquitin-conjugating enzyme complex UBC13–UEV1A to catalyze the synthesis of K63-linked polyubiquitin chains (polyUb) (Deng et al., 2000). K63-polyUb recruits the TAK1 kinase complex by binding to the regulatory subunit TAB2, and this recruitment facilitates the autophosphorylation and activation of TAK1 (Kanayama et al., 2004; Wang et al., 2001). PolyUb also recruits the IKK complex by binding to NEMO, thereby promoting the phosphorylation of IKKβ by TAK1 (Ea et al., 2006; Wu et al., 2006; Xia et al., 2009). The activated IKK complex then phosphorylates IκB proteins, leading to their degradation and subsequent activation of NF-κB (Liu and Chen, 2011).

TNFα binds to and activates TNF receptor (TNFR1), which recruits adaptor proteins including tumor necrosis factor receptor type 1-associated DEATH domain protein (TRADD) and receptor-interacting serine/threonine-protein kinase 1 (RIPK1) (Wertz and Dixit, 2010). TNFR1 also recruits and activates E3 ligases including TNF receptor associated factor 2 (TRAF2), TRAF5, the cellular inhibitors of apoptosis (cIAP1 and cIAP2), and linear ubiquitin chain assembly complex (LUBAC), which consists of HOIP, HOIL-1, and Sharpin. The TRAF and cIAP proteins function as ubiquitin ligases that catalyze polyubiquitination including K63 polyubiquitin chain synthesis, whereas LUBAC catalyzes linear polyubiquitination in which the C-terminus of one ubiquitin is linked to the N-terminal methionine (M1) of the next ubiquitin. Similarly to the IL-1β pathway, K63- or M1- linked polyUb activates TAK1 and IKK complexes, leading to NF-κB activation (Liu and Chen, 2011; Wertz and Dixit, 2010).

**Figure 1.**
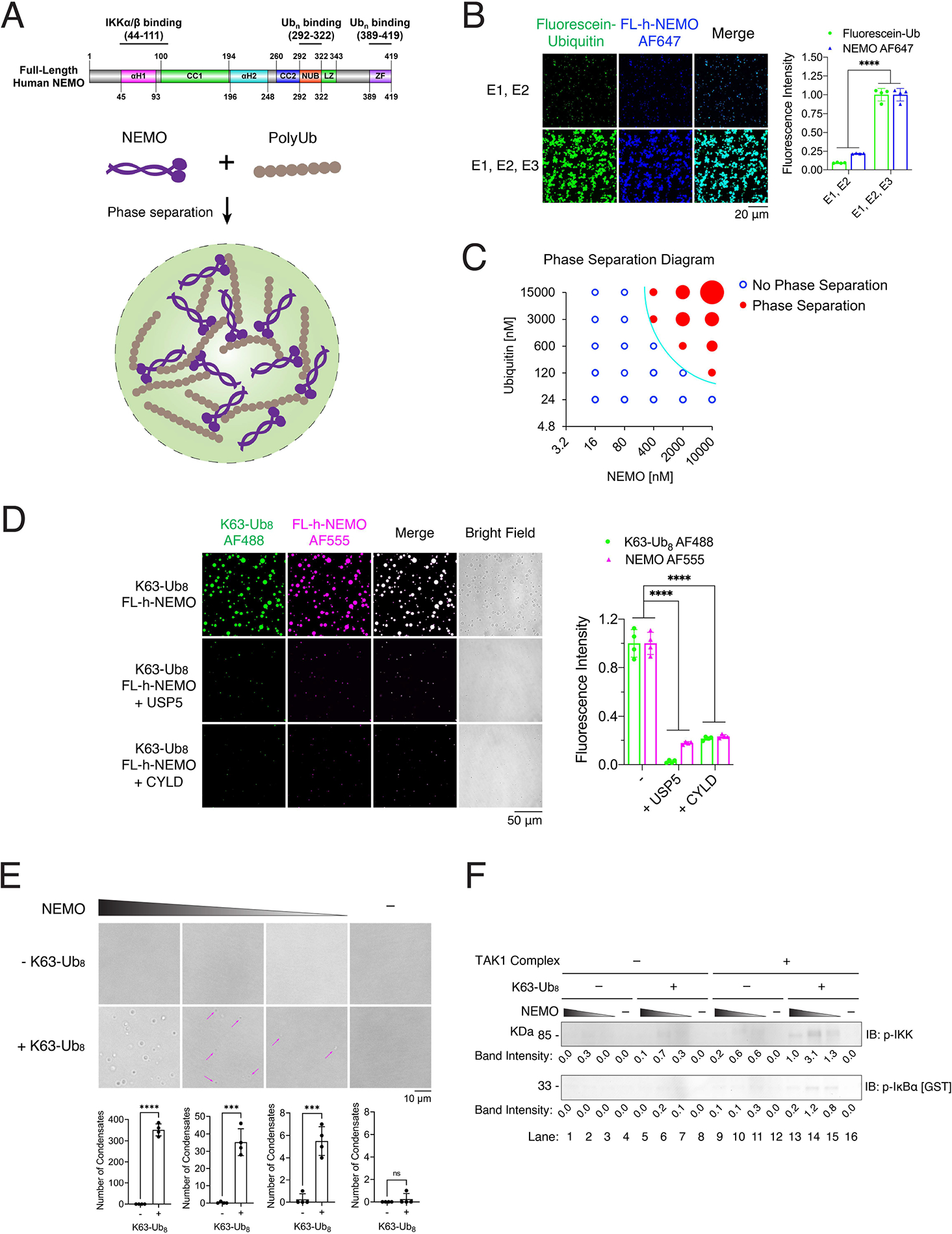
Polyubiquitin chains induce phase separation of NEMO (A) Schematic of NEMO structural domains and a model of polyUb-induced phase separation of NEMO. (B) Left panel: representative fluorescent images of NEMO liquid droplets induced by enzymatically synthesized K63-polyUb. Liquid droplets formed after mixing of 6 μM human FL-NEMO (3% was labeled with Alexa Fluor 555) with 14.5 μM ubiquitin (3% was labeled with Fluorescein) in a reaction mixture containing E1 and UBC13–UEV1A (E2) in the presence or absence of TRAF6 (E3) for an hour at 37°C. Small droplets were visible in the absence of TRAF6 because Ubc13/Uev1A was capable of synthesizing small amounts of short K63-polyUb without TRAF6 (Figure S1C). Right panel: quantification of fluorescence intensity of liquid droplets. (C) Phase separation diagram of human FL-NEMO and ubiquitin at indicated concentrations. PolyUb chains were synthesized by mixing recombinant NEMO protein with ubiquitin at indicated concentrations, 85 nM E1, 333 nM UBC13-UEV1A, and 156 nM TRAF6 for an hour at 37°C in a buffer containing 20 mM Tris-HCl, pH 7.5, 5 mM MgCl_2_, 5 mM ATP, 2 mM DTT and 0.2 mg/ml BSA. Blue empty circles: no liquid droplets formed; Red solid circles: liquid droplets formed, and sizes of circles illustrate the sizes of liquid droplets (Figure S1D). (D) Left panel: representative fluorescent images of NEMO liquid droplets induced by K63-Ub_8_ in the absence or presence of USP5 or CYLD. Liquid droplets formed after mixing of 6 μM human FL-NEMO (3% was labeled with Alexa 555) with 2.25 μM K63-Ub_8_ (3% was labeled with Alexa 488) for an hour at 37°C. Right panel: quantification of fluorescence intensity of liquid droplets. (E) Upper panel: representative bright field images of NEMO liquid droplets induced by K63-Ub_8_. Liquid droplets formed after mixing of 1 μM K63-Ub_8_ with human FL-NEMO (1000, 100, 10, and 0 nM respectively) for an hour at 37°C. Magenta arrows indicate the liquid droplets. Lower panel: quantification of the numbers of liquid droplets. (F) IKK activation by NEMO and K63-Ub_8_. IKK isolated from the NEMO-deficient 1.3E2 cells was incubated in the presence or absence of the TAK1 complex, 1 μM K63-Ub_8_ and NEMO (1000, 100, 10, and 0 nM respectively) in a reaction mixture (10 μl) containing GST-IκBα. After incubation at 30 °C for 1 h, IKK activity was analyzed by immunoblotting using an antibody against phospho-IKKα/β or phospho-IκBα. Numbers below the bands denote the quantification of bands. Data shown in (B), (D) and (E) are the means ± SD. n = 4 areas. In (B) and (D), two-way analysis of variance (ANOVA). In (E), unpaired t test. n.s., P > 0.0332; ***P < 0.0002; ****P < 0.0001. See also Figure S1 and S2.

NEMO contains an IKKα/IKKβ binding domain at the N-terminus and two ubiquitin binding domains (UBDs) at the C-terminus. The UBDs include a NEMO ubiquitin binding (NUB) domain (also known as UBAN) and a zinc finger (ZF) domain, both of which bind to K63-linked polyUb as well as linear polyUb (Figure 1A) (Napetschnig and Wu, 2013). NEMO forms a head-to-head dimer; thus, each dimer of NEMO has at least four polyUb binding sites. In turn, each polyUb can potentially bind to multiple NEMO proteins, depending on the polyUb chain lengths. Previous studies have shown that polyUb binding by NEMO is important for IKK activation (Ea *et al.*, 2006; Wu *et al.*, 2006), but the underlying biochemical mechanism has remained unknown. NEMO has also been shown to form puncta structures in cells stimulated with TNFα or IL-1β (Cruz et al., 2021; Tarantino et al., 2014), but the nature of such puncta and their role in IKK activation have not been characterized.

Here we show that polyUb binding to NEMO robustly induced the formation of liquid-like droplets both in vitro and in cells. This liquid phase condensation of NEMO is driven by multivalent interactions between NEMO and polyUb. Both the NUB and ZF ubiquitin-binding domains are important for NEMO phase separation and IKK activation. NEMO mutations with diminished polyUb binding, which are linked to human immunodeficiency, impaired NEMO phase separation and NF-κB activation. These results demonstrate that polyUb-induced phase separation of NEMO underlies IKK activation.

## RESULTS

### Polyubiquitin chains induce phase separation of NEMO

We have previously shown that TRAF6 is a ubiquitin E3 ligase that functions together with the ubiquitin activating enzyme (E1) and ubiquitin conjugating enzyme (E2) complex UBC13–UEV1A to synthesize K63-linked polyUb chains, which then activate the TAK1 and IKK complexes (Deng *et al.*, 2000; Wang *et al.*, 2001; Xia *et al.*, 2009). To test whether K63-polyUb chains induce phase separation of NEMO in vitro, we incubated Alexa Fluor 647 (AF647) -labeled human full-length NEMO protein with a reaction mixture containing fluorescein-labeled ubiquitin, E1, UBC13–UEV1A in the presence or absence of partially purified TRAF6 (Figure 1A, 1B and S1A–C). This reaction has been shown to predominantly synthesize unanchored K63-polyUb chains which bind non-covalently to NEMO (Xia *et al.*, 2009) (Figure S1C). Imaging of the reaction mixtures revealed micrometer-sized liquid droplets that contained both NEMO and ubiquitin (Figure 1B). Supplementing TRAF6 in the enzymatic reaction led to the synthesis of more and longer K63-polyUb (Figure S1C), and NEMO formed more numerous and larger liquid droplets in the presence of TRAF6 (Figure 1B). The formation of such liquid droplets was dependent on the concentrations of both NEMO and ubiquitin, and larger liquid droplets formed as the concentrations increased (Figure 1C and S1D). In this reaction system, two processes occurred: polyubiquitination and liquid droplet formation. To uncouple these two steps, we incubated purified NEMO with a K63 polyUb chain consisting of eight ubiquitin (K63-Ub_8_). Both NEMO and K63-Ub_8_ phase separated into liquid droplets, which were abolished by treatment with CYLD, a deubiquitination enzyme known to specifically cleave K63-linked and M1-linked polyUb, or USP5 (also known as isopeptidase T), an enzyme known to specifically cleave unanchored polyUb from the C-terminus (Figure 1D and S2) (Xia *et al.*, 2009). These results demonstrate that K63-polyUb chains induce liquid phase condensation of NEMO in vitro.

To test if NEMO condensates induced by polyUb activate IKK in vitro, we reconstituted IKK activation in vitro using purified proteins, including NEMO, K63-Ub_8_, TAK1-TAB1-TAB2 kinase complex, IKK complex (lacking NEMO), and GST-IκBα. These proteins were incubated together with a buffer containing ATP, and the activation of IKK was analysed by immunoblotting with antibodies against phosphorylated IKKα/β (p-IKK) and p-IκBα. In the presence of NEMO, K63-Ub_8_ and the TAK1 kinase complex, IKK was activated as indicated by phosphorylation of IKKα/β and IκBα (Figure 1F). The IKK activities correlated with the formation of NEMO condensates (Figure 1E); however, at higher NEMO concentration, the IKK activity decreased presumably because excess NEMO that is not in the IKK complex competed away the ubiquitin chains from the IKK complex. These results indicate that polyUb binding induces NEMO phase separation as well as IKK activation in vitro.

**Figure 2.**
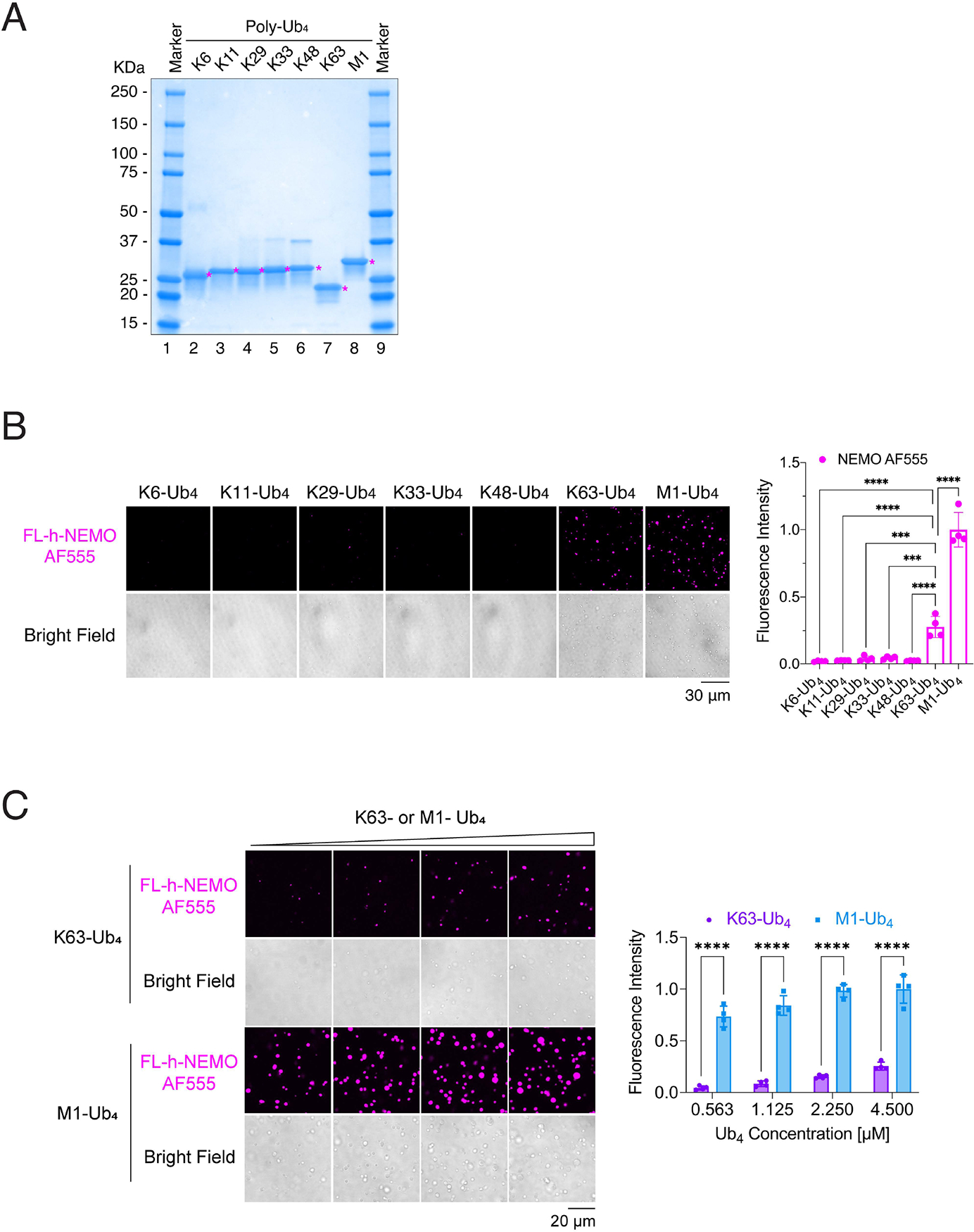
NEMO phase separation is specifically induced by K63-linked and linear polyubiquitin chains (A) Coomassie blue staining of Ub_4_ linked through K6, K11, K29, K33, K48, K63 or M1. Asterisks indicate the protein of interest (Ub_4_). (B) Left panel: representative fluorescent images of NEMO liquid droplets induced by Ub_4_ linked through K6, K11, K29, K33, K48, K63, and M1. Liquid droplets formed after mixing of 6 μM human FL-NEMO (3% was labeled with Alexa 555) with 4.5 μM Ub_4_ of different linkages for 30 minutes at 37°C. Right panel: quantification of fluorescence intensity of liquid droplets. (C) Left panel: representative fluorescent images of NEMO liquid droplets induced by K63- or M1- Ub_4_ at different concentrations. Liquid droplets formed after mixing of 6 μM human FL-NEMO (3% was labeled with Alexa 555) with indicated concentrations of K63- or M1- Ub_4_ for 45 minutes at 37°C. Right panel: quantification of fluorescence intensity of liquid droplets. Data shown in (B) and (C) are means ± SD. n = 4 areas. In (B), one-way analysis of variance (ANOVA); in (C), two-way analysis of variance (ANOVA). ***P < 0.0002; ****P < 0.0001. See also Figure S3.

To determine if polyUb of different linkages could also induce NEMO phase separation, we incubated Alexa Fluor 555 (AF555) -labeled NEMO with purified Ub_4_ linked through K6, K11, K29, K33, K48, K63 or M1 (Figure 2A and 2B). Only M1- and K63-linked Ub_4_ were able to induce NEMO phase separation, consistent with previous findings that only these polyUb bind to NEMO (Figure 2B) (Ea *et al.*, 2006; Rahighi et al., 2009; Wu *et al.*, 2006). M1-linked Ub_4_ induced more robust NEMO phase separation than K63-Ub_4_ (Figure 2C), consistent with previous reports that short M1-linked ubiquitin chains have higher affinity for NEMO than short K63-linked ubiquitin chains (Rahighi *et al.*, 2009). Further confirming these results, incubation of AF555-labeled NEMO with purified Ub_2–7_ linked through K48, K63 or M1 showed that only M1- and K63- linked Ub_2–7_ were able to induce NEMO liquid droplet formation (Figure S3A and S3B; note that K63-linked Ub_2–7_ appeared to have some minor smears that may represent impurity; however, this does not affect the conclusion because K63-Ub_4_ induced robust phase separation of NEMO, Figure 2C).

The binding of TNFα to its receptor leads to the activation of multiple E3 ligases, including TRAF2/5, cIAP1/2 and LUBAC, to synthesize K63- or linear polyUb chains (Liu and Chen, 2011; Wertz and Dixit, 2010). To test the potential synergism between these two ubiquitin chain types, we tested if NEMO undergoes phase separation with a mixture of K63 and M1 polyUb. The mixed ubiquitin chains induced phase separation of NEMO but it was not more robust than the M1 polyUb alone (Figure S3C).

Hybrid K63/M1 and K48/K63 chains are formed in cells in response to activation of several receptors, including TNFR1 and IL-1R (Emmerich et al., 2016; Emmerich et al., 2013; Ohtake et al., 2016). To test if NEMO phase separation can be induced by K48/K63 and K63/M1 hybrid polyUb, we enzymatically synthesized the hybrid polyUb chains by mixing of di-ubiquitin (K48, M1, or K63) with E1, E2 (UBC13/UEV1A), and E3 (TRAF6) in a reaction buffer containing ATP (Figure S3D). NEMO also underwent phase condensation with K48/K63 or K63/M1 hybrid polyUb although the hybrid ubiquitin chains did not appear to induce more robust phase separation of NEMO (Figure S3E).

### NEMO forms liquid-like condensates in cells stimulated with IL-1β or TNFα

**Figure 3.**
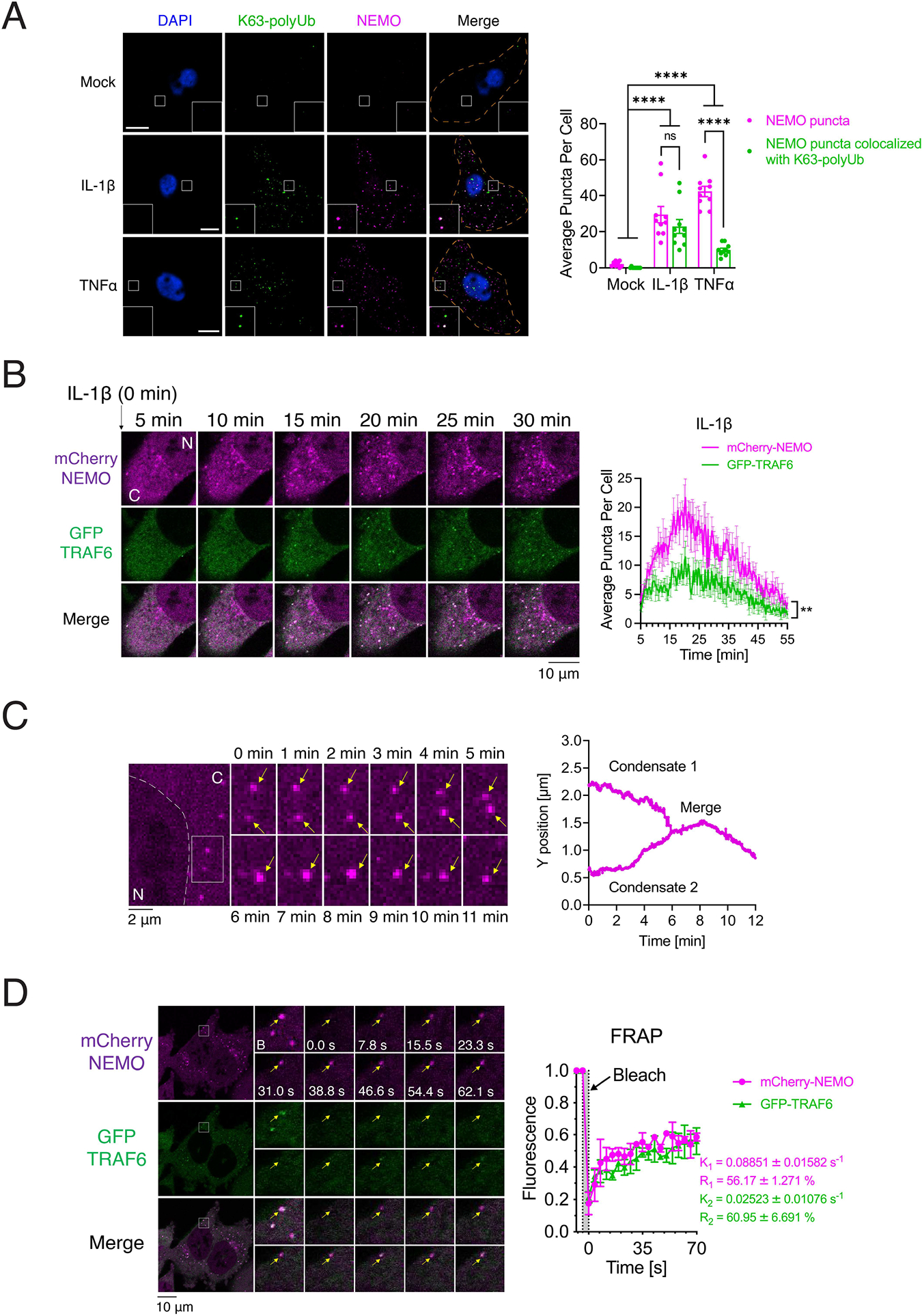
NEMO forms liquid-like condensates in cells in response to stimulation (A) Left panel: Immunofluorescence staining of K63-linked polyubiquitin chains in U2OS^mCherry-NEMO Knock-in^ cells stimulated with either IL-1β or TNFα. Scale bars, 10 μm. Right panel: quantification of average numbers of NEMO puncta and those containing both NEMO and K63-polyUb per cell. Shown are means ± SEM. n = 10 cells. Two-way analysis of variance (ANOVA). n.s., P > 0.0332; ****P < 0.0001. (B) Left panel: representative live cell images of NEMO condensates in HCT116^NEMO KO^-mCherry-NEMO/GFP-TRAF6 cells stimulated with IL-1β. N: nucleus; C: cytoplasm. Right panel: quantification of average numbers of condensates containing NEMO or TRAF6 per cell over the time course. Shown are means ± SEM. n = 4 cells. **P < 0.0021, one-way analysis of variance (ANOVA). (C) Left panel: time-lapse micrographs of NEMO condensate formation and fusion (time 0 represents 20 min after adding IL-1β). Arrows indicate condensates. The fusion events existed in all three fields examined. N: nucleus; C: cytoplasm. Right panel: tracking the movement and fusion (y position vs time) of two NEMO condensates. (D) Left panel: representative micrographs of NEMO condensates before and after photobleaching (arrow, bleach site). NEMO condensates formed in HCT116^NEMO KO^-mCherry-NEMO/GFP-TRAF6 cells stimulated by IL-1β. Right panel: quantification of FRAP of NEMO puncta over a 70-s time course. K, exponential constant; R, normalized plateau after fluorescence recovery. Shown are means ± SD. n = 3 NEMO condensates. See also Figure S4, S5 and S6.

We next examined if NEMO and K63-polyUb form liquid condensates in cells. We employed the CRISPR-Cas9 technology to knock in a mCherry tag at the NEMO gene locus in the human osteosarcoma cell line U2OS (U2OS^mCherry-NEMO Knock-in^ cells hereafter) (Figure S4A–B). The mCherry-NEMO was expressed in the cells at a level similar to that of endogenous NEMO and supported robust IκBα phosphorylation and degradation in response to stimulation with IL-1β or TNFα (Figure S4B). Immunostaining with an antibody against K63-polyUb showed that NEMO and K63-polyUb co-localized in cytoplasmic puncta of the stimulated cells (Figure 3A). To observe the dynamics of NEMO puncta in the cytoplasm, we performed live cell imaging of NEMO and TRAF6 in the human colorectal carcinoma cell line HCT116 in which endogenous NEMO was knocked out by CRISPR and reconstituted with mCherry-tagged NEMO and green fluorescent protein (GFP) tagged TRAF6. Upon IL-1β stimulation, NEMO condensates appeared within 5 min, and the condensate numbers peaked at around 20 min, then declined, and disappeared at around 55 min (Figure 3B). TRAF6 was detected in a fraction of NEMO condensates (Figure 3B). Upon TNFα stimulation, NEMO condensates appeared within 2 min, and the condensate numbers peaked at around 10 min, then declined, and disappeared at around 27 min (Figure S5A). In contrast to IL-1β stimulation, TRAF6 was not detected in the NEMO condensates induced by TNFα (Figure S5A), consistent with TRAF6 not being involved in the TNFα pathway (Liu and Chen, 2011). To test if other E3 ligases co-localize with the NEMO puncta, we performed immunofluorescent staining of endogenous NEMO and RNF31 (also known as HOIP, one of the three components of LUBAC), cIAP1 or TRAF2 in U2OS cells. In response to stimulation with IL-1β or TNFα, NEMO formed condensates in stimulated cells and partially colocalized with RNF31. cIAP1 and TRAF2 partially co-localized with NEMO condensates in response to stimulation with TNFα, but not IL-1β (Figure S6). The percentages of cells that contained NEMO puncta after stimulation with IL-1β or TNFα were > 90% for all examined cells.

After stimulation with IL-1β or TNFα, the NEMO foci exhibited liquid-like properties, as demonstrated by the ability of two foci to fuse with each other (Figure 3C). Furthermore, fluorescence recovery after photobleaching (FRAP) experiments showed that NEMO in the foci displayed half maximal fluorescence recovery in ~11 seconds (K_1_ = 0.08851 ± 0.01582 s^−1^) in cells stimulated with IL-1β, whereas TRAF6 achieved half maximal recovery in ~40 seconds (K_2_ = 0.02523 ± 0.01076 s^−1^) (Figure 3D). In response to TNFα stimulation, NEMO in the foci achieved half maximal fluorescence recovery in ~20 seconds (K = 0.04980 ± 0.01133 s^−1^) (Figure S5B). These results indicate that NEMO exhibited dynamic liquid-like behavior in the cytoplasmic foci.

### IKK is activated within the NEMO condensates

**Figure 4.**
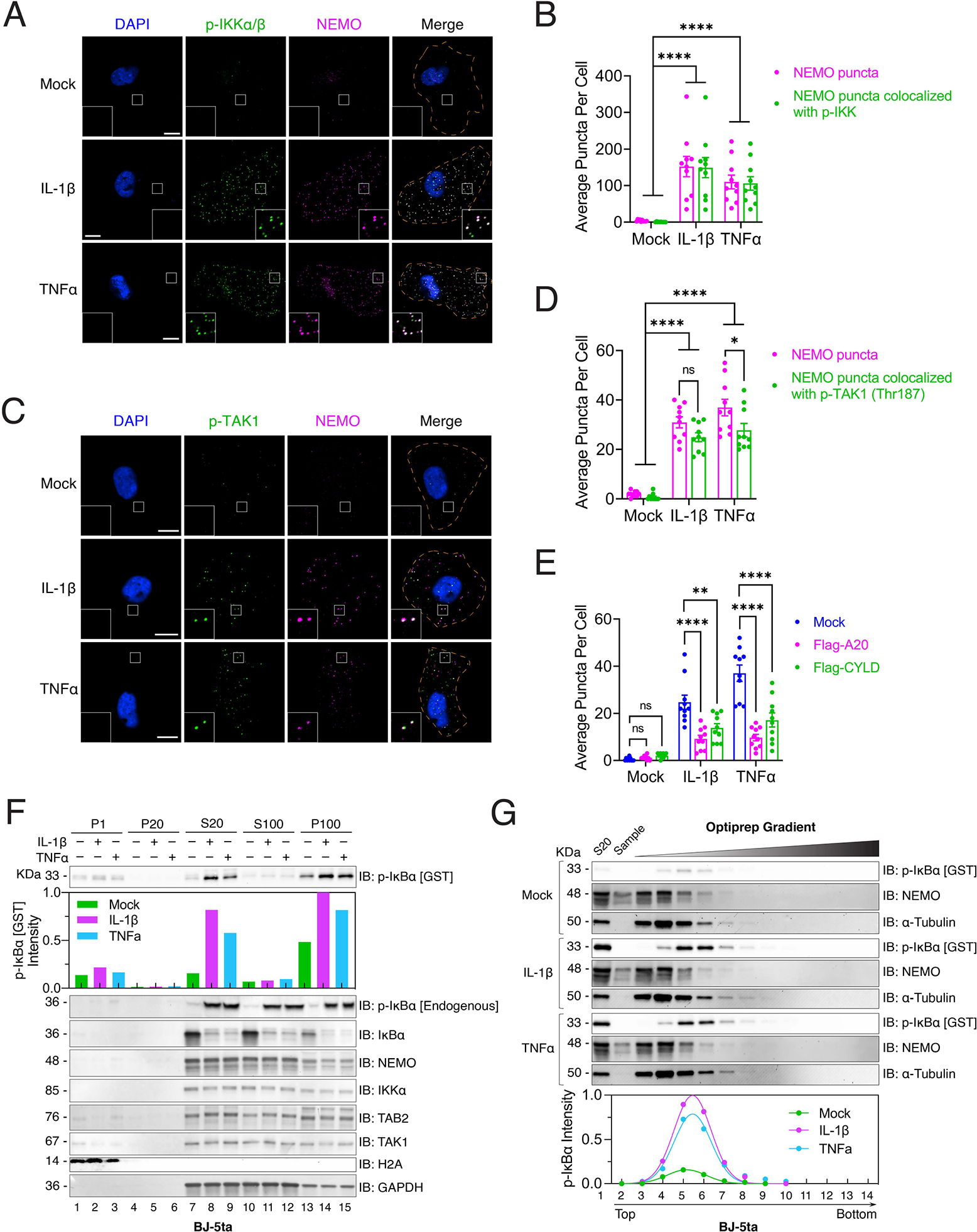
Activated IKK is contained within the NEMO condensates in cells stimulated with IL-1β or TNFα (A) Immunofluorescence staining of NEMO and p-IKKα/β in BJ-5ta cells stimulated with IL-1β or TNFα. Scale bars, 10 μm. (B) Quantification of average numbers of NEMO puncta and those containing both NEMO and p-IKKα/β per cell shown in (A). (C) Immunofluorescence staining of p-TAK1 (Thr187) in U2OS^mCherry-NEMO Knock-in^ cells stimulated with either IL-1β or TNFα. Scale bars, 10 μm. (D) Quantification of average number of NEMO puncta and those containing both NEMO and p-TAK1 (Thr187) per cell shown in (C). (E) Quantification of average number of NEMO puncta per cell in U2OS^mCherry-NEMO Knock-in^ cells transiently overexpressing Flag-A20 or Flag-CYLD. The representative images used for the quantification are shown in Figure S8A. (F) Subcellular fractionation and measurement of IKK activity. BJ-5ta cells stimulated with IL-1β or TNFα were fractionated by differential centrifugation as depicted in Figure S10A. Fractions were incubated with recombinant GST-IκBα in a kinase reaction buffer, followed by immunoblotting with an antibody against p-IκBα. Bar plot represents the quantification of p-GST-IκBα. Fractions were also analyzed by immunoblotting (IB) with antibodies against endogenous p-IκBα, IκBα, NEMO, IKKα, TAB2, TAK1, histone H2A (nuclear marker), or glyceraldehyde-3-phosphate dehydrogenase (GAPDH) (cytoplasmic marker). (G) The S20 fractions from (F) were further separated by OptiPrep gradient ultracentrifugation, and IKK activity in different fractions was measured as in (F). Plot shows quantification of IKK activity after OptiPrep gradient ultracentrifugation. Dots represent quantification of p-GST-IκBα and curves represent the dots fitting by using a gaussian distribution model (Least square fit). Data shown in (B), (D) and (E) are means ± SEM. n = 10 cells. Two-way analysis of variance (ANOVA). n.s., P > 0.0332; *P < 0.0332; **P < 0.0021; ****P < 0.0001. See also Figure S7, S8, S9, S10 and S11.

To test if the NEMO condensates contained activated kinases, we performed immunofluorescent staining of endogenous NEMO and p-IKKα/β in BJ-5ta cells in response to IL-1β or TNFα stimulation. NEMO formed condensates in stimulated cells and the active IKK (p-IKKα/β) co-localized with NEMO condensates (Figure 4A–B). Similarly, TAB2, TAK1 and p-TAK1 (TAK1 phosphorylated at Thr187) also co-localized with NEMO condensates in cells stimulated with IL-1β or TNFα (Figure 4C–D; Figure S7). These results demonstrate that the activated IKK and TAK1 are contained within the NEMO condensates in cells stimulated with IL-1β or TNFα. Overexpression of the deubiquitination enzymes CYLD and A20, which are known to inhibit NF-κB, reduced the numbers of NEMO condensates and inhibited IKK activation in U2OS^mCherry-NEMO Knock-in^ cells stimulated with IL-1β or TNFα (Figure 4E and S8), indicating that polyUb chains are required for the formation of NEMO condensates. We also observed that IRAK1 but not mCherry-IL-1β co-localized with NEMO condensates in U2OS cells, whereas mCherry-TNFα co-localized with NEMO condensates (Figure S9). These results are consistent with previous reports showing that the IKK complex is associated with the TNF receptor but not IL-1 receptor after stimulation (Tarantino *et al.*, 2014).

To further determine the function of NEMO condensates, we stimulated human fibroblast BJ-5ta cells with IL-1β or TNFα, prepared subcellular fractions by differential centrifugation (Figure S10A) and measured IKK activity in each fraction with an in vitro kinase assay using GST-IκBα as a substrate (Figure S10B). Most IKK activity was present in the supernatant obtained after centrifugation at 20,000 × g (designated S20) (Figure 4F). Upon further centrifugation of S20 at 100,000 × g, the majority of IKK activity was detected in the pellet (designated P100; Figure 4F and S10E). Fluorescent imaging of the fractions from U2OS^mCherry-NEMO Knock-in^ cells revealed that NEMO condensates were enriched in p100 from cells stimulated with IL-1β or TNFα (Figure S11). Optiprep (Iodixanol) density gradient ultracentrifugation of S20 showed that the IKK activity was mainly detected in fractions 5–6 (~10% iodixanol), whereas NEMO and the cytoplasmic protein marker tubulin were mainly present in fraction 4 (~5% iodixanol) (Figure 4G). Thus, only a fraction of the NEMO/IKK complex (estimated at ~20%, see methods) was catalytically active, and this fraction was associated with the NEMO condensates. Similar results were obtained using U2OS cells (Figure S10C–D, F).

### Multivalent interactions between NEMO and polyUb drive phase separation

**Figure 5.**
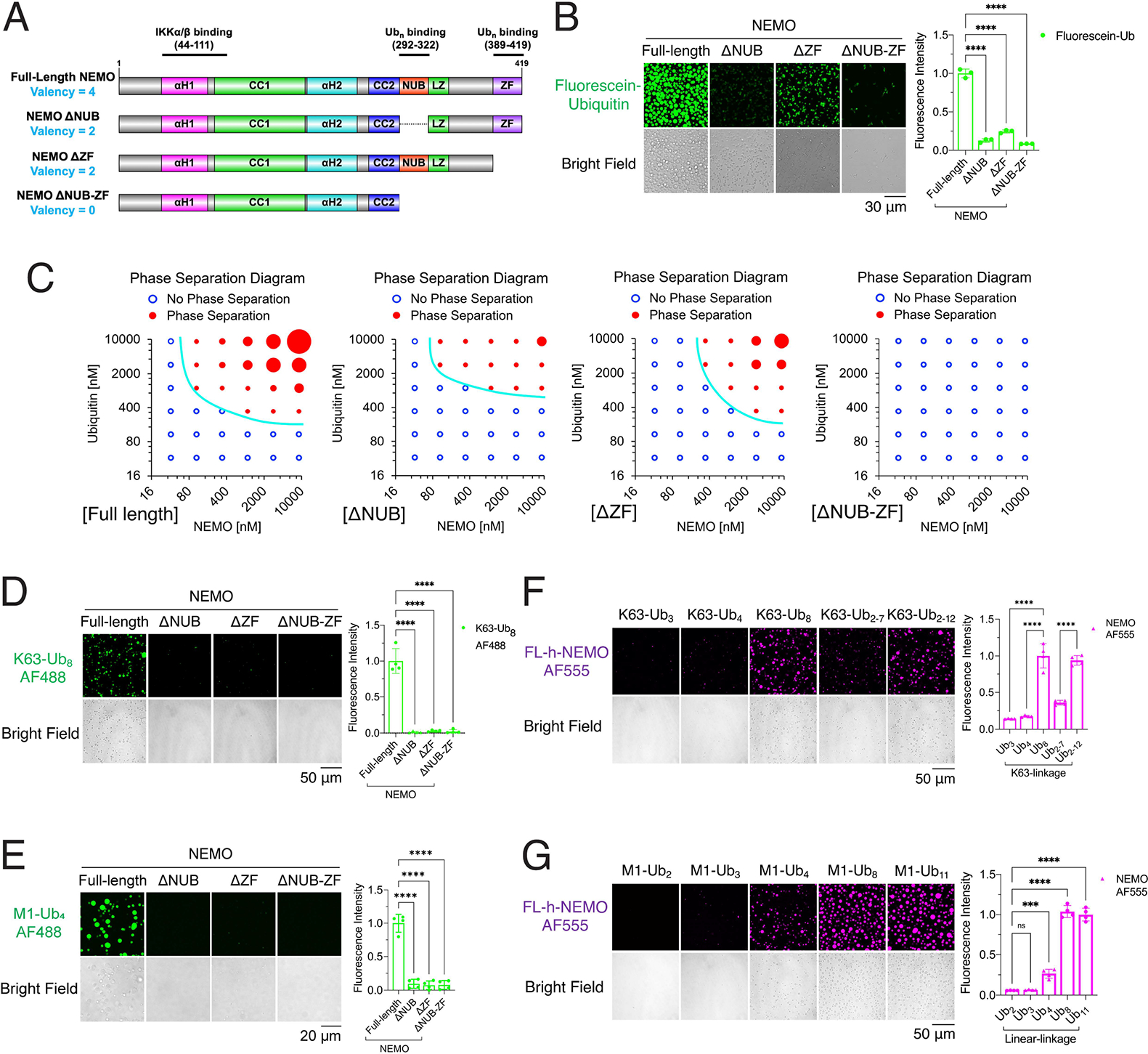
Multivalent interactions between NEMO and polyUb drive their liquid phase separation (A) Schematic of valencies of ubiquitin binding by NEMO and its ubiquitin-binding mutants. (B) Left panel: representative images of phase separation by mixing full-length NEMO or its mutants (ΔNUB, ΔZF and ΔNUB-ZF) with K63-polyUb chains synthesized in a reaction mixture containing E1, UBC13/UEV1A, TRAF6, ubiquitin and fluorescein ubiquitin. Right panel: quantification of fluorescence intensity of liquid droplets. Shown are means ± SD. n = 3 areas. (C) Phase separation diagrams of varying concentrations of NEMO or its mutants incubated with K63-polyUb chains synthesized in a reaction mixture containing varying concentrations of ubiquitin. Blue empty circles: no liquid droplets formed; Red solid circles: liquid droplets formed, and the circle sizes illustrate the sizes of liquid droplets. Representative images of the liquid droplets are shown in Figure S12B. (D) Left panel: representative images of phase separation by mixing K63-Ub_8_ with NEMO or its mutants as indicated. Right panel: quantification of fluorescence intensity of liquid droplets. Shown are means ± SD. n = 4 areas. (E) Left panel: representative images of phase separation by mixing M1-Ub_4_ with NEMO or its mutants as indicated. Right panel: quantification of fluorescence intensity of liquid droplets. Shown are means ± SD. n = 4 areas. (F) Left panel: representative images of phase separation by mixing full-length human NEMO with K63-polyUb of different lengths as indicated. Right panel: quantification of fluorescence intensity of liquid droplets. Shown are means ± SD. n = 4 areas. (G) Left panel: representative images of phase separation by mixing full-length human NEMO with linear polyUb of different lengths as indicated. Right panel: fluorescence intensity quantification of liquid droplets. Shown are means ± SD. n = 4 areas. In (B), (D), (E), (F) and (G): One-way analysis of variance (ANOVA); n.s., P > 0.0332; ***, P < 0.0002; ****, P < 0.0001. See also Figure S12.

Multivalent interactions drive liquid phase separation (Banani et al., 2017; Li et al., 2012). NEMO contains two polyUb-binding sites: NEMO ubiquitin binding (NUB) domain (human: amino acid 292–322) and a zinc finger (ZF) domain (human: amino acid 389–419; Figure 5A). Crystal structures of NEMO (CC2-LZ region) show that it forms a head-to-head dimer mediated by hydrophobic residues and aliphatic portions of charged residues (Napetschnig and Wu, 2013). The NEMO dimer contains at least four binding sites for polyUb (valency = 4) (Figure 5A). Similarly, long polyUb has more binding sites for NEMO than short polyUb. To test whether the multivalent interactions between NEMO and polyUb drive their phase separation, we incubated full-length (FL) -NEMO or NEMO lacking one or both Ub-binding domains (NEMO-ΔNUB, NEMO-ΔZF, or NEMO-ΔNUB-ZF) with K63-polyUb of different lengths (Figure S12A). FL-NEMO formed more numerous and larger liquid droplets with polyUb synthesized in a reaction mixture containing fluorescein-labeled ubiquitin, E1, UBC13–UEV1A and TRAF6, than the NEMO mutants with compromised polyUb binding (Figure 5B). Furthermore, FL-NEMO required lower concentrations of ubiquitin or NEMO to form liquid droplets than NEMO-ΔNUB, NEMO-ΔZF, or NEMO-ΔNUB-ZF (Figure 5C and S12B). Besides, FL-NEMO formed liquid droplets with K63-Ub_8_ and M1-Ub_4_ robustly whereas the NEMO mutants with compromised polyUb binding had markedly diminished ability to form liquid droplets (Figure 5D & E). Moreover, longer K63-polyUb and M1-polyUb formed more numerous and larger liquid droplets with full-length human NEMO than shorter K63- or M1- polyUb (Figure 5F & G and S12C & D). These results demonstrate that multivalent interactions between NEMO and polyUb drive the liquid phase separation of NEMO with polyUb.

### Multivalent interactions between NEMO and polyUb led to NEMO phase separation and IKK activation in cells

**Figure 6.**
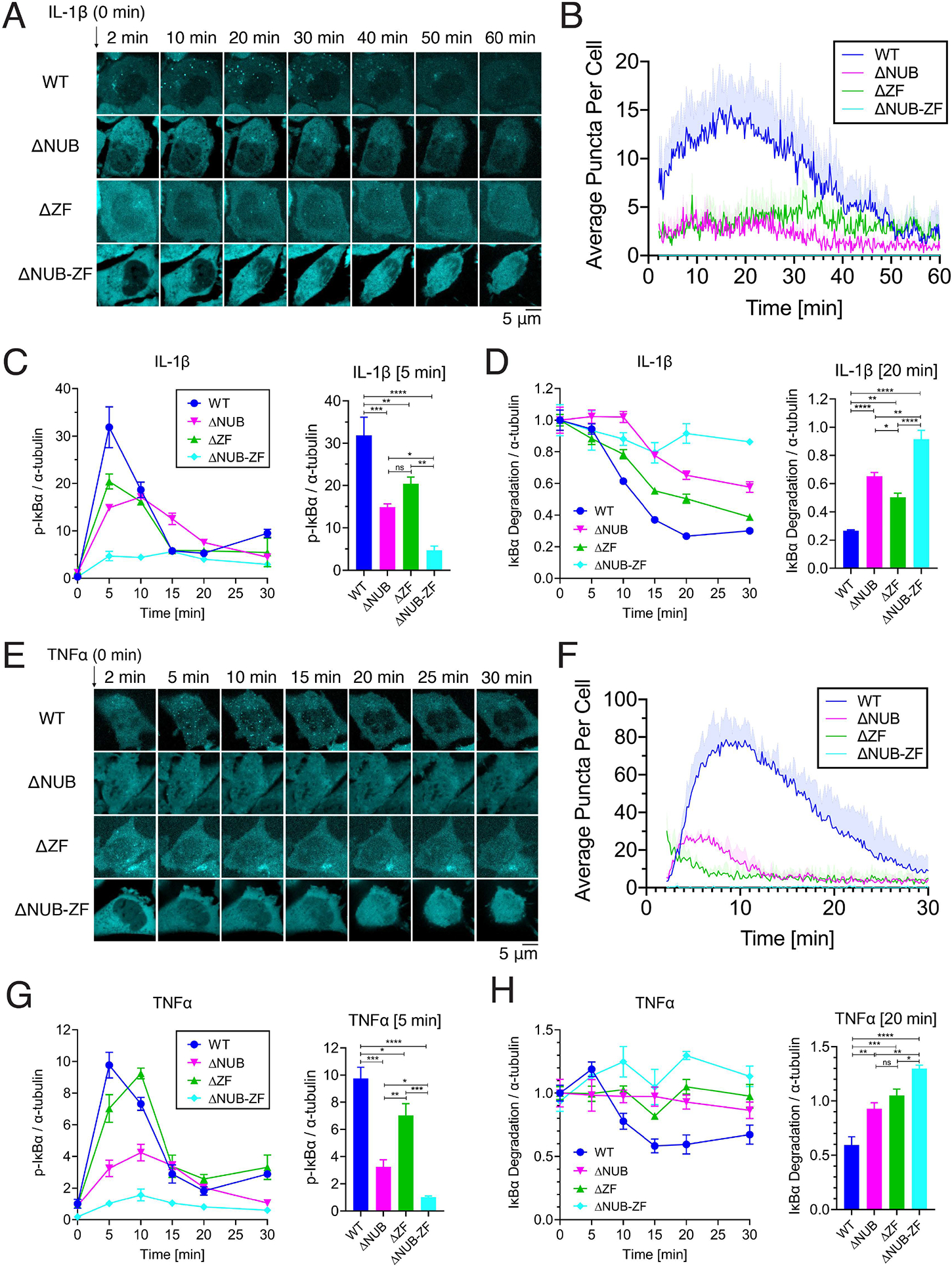
Multivalent interactions between NEMO and polyUb lead to NEMO phase separation and IKK activation in cells (A) Representative live cell images of NEMO condensate formation upon IL-1β stimulation in HCT116^NEMO KO^- GFP-TRAF6 cells reconstituted with mCherry-tagged human NEMO or indicated mutants. (B) Quantification of average NEMO condensates per cell versus time in (A). Shown are means ± SEM. n = 4 cells. (C) Left panel: Immunoblotting of p-IκBα in response to IL-1β stimulation in the same cell lines shown in (A). Right panel: phosphorylation of IκBα in cells stimulated with IL-1β for 5 min. (D) Left panel: similar to (C) except that an antibody against IκBα was used and cells were stimulated with IL-1β for 20 min (right panel). (E) Representative live cell images of NEMO condensate formation upon TNFα stimulation in the same cell lines as in (A). (F) Quantification of average NEMO condensates per cell versus time in (E). Shown are means ± SD. n = 3 cells. (G) and (H), similar to (C) and (D) respectively, except that cells were stimulated with TNFα for the indicated time. (C), (D), (G), and (H): Shown are means ± SEM. n = 3 biological replicates. One-way analysis of variance (ANOVA); n.s., P > 0.0332; *, P < 0.0332; **, P < 0.0021; ***, P < 0.0002; ****, P < 0.0001. See also Figure S13 and S14, Movie S1 and S2.

To test the effect of polyUb binding on NEMO phase separation and IKK activation in cells, we reconstituted NEMO-deficient HCT116 cells with mCherry-tagged wild type NEMO, NEMO–ΔNUB, NEMO–ΔZF, or NEMO–ΔNUB-ZF (Figure 5A, 6A and S13A). Live cell imaging after IL-1β or TNFα stimulation showed that cells containing wild type NEMO formed more numerous puncta than cells containing NEMO–ΔNUB or NEMO–ΔZF, while no puncta were observed in cells containing NEMO–ΔNUB-ZF (Figure 6A, B, E and F; Movie S1 and S2). We also examined the activation of IKK complex in cells by quantifying the phosphorylation and degradation of IκBα after IL-1β or TNFα treatment. Cells expressing wild-type NEMO exhibited stronger phosphorylation and degradation of IκBα than cells containing NEMO–ΔNUB or NEMO–ΔZF, whereas the cells expressing NEMO–ΔNUB-ZF were completely defective in phosphorylation or degradation of IκBα (Figure 6C, D, G and H, S13B, S14). These results demonstrate that polyUb binding is required for NEMO phase separation as well as IKK activation.

### Disease-associated mutations of NEMO impair its phase separation with polyubiquitin chains

**Figure 7.**
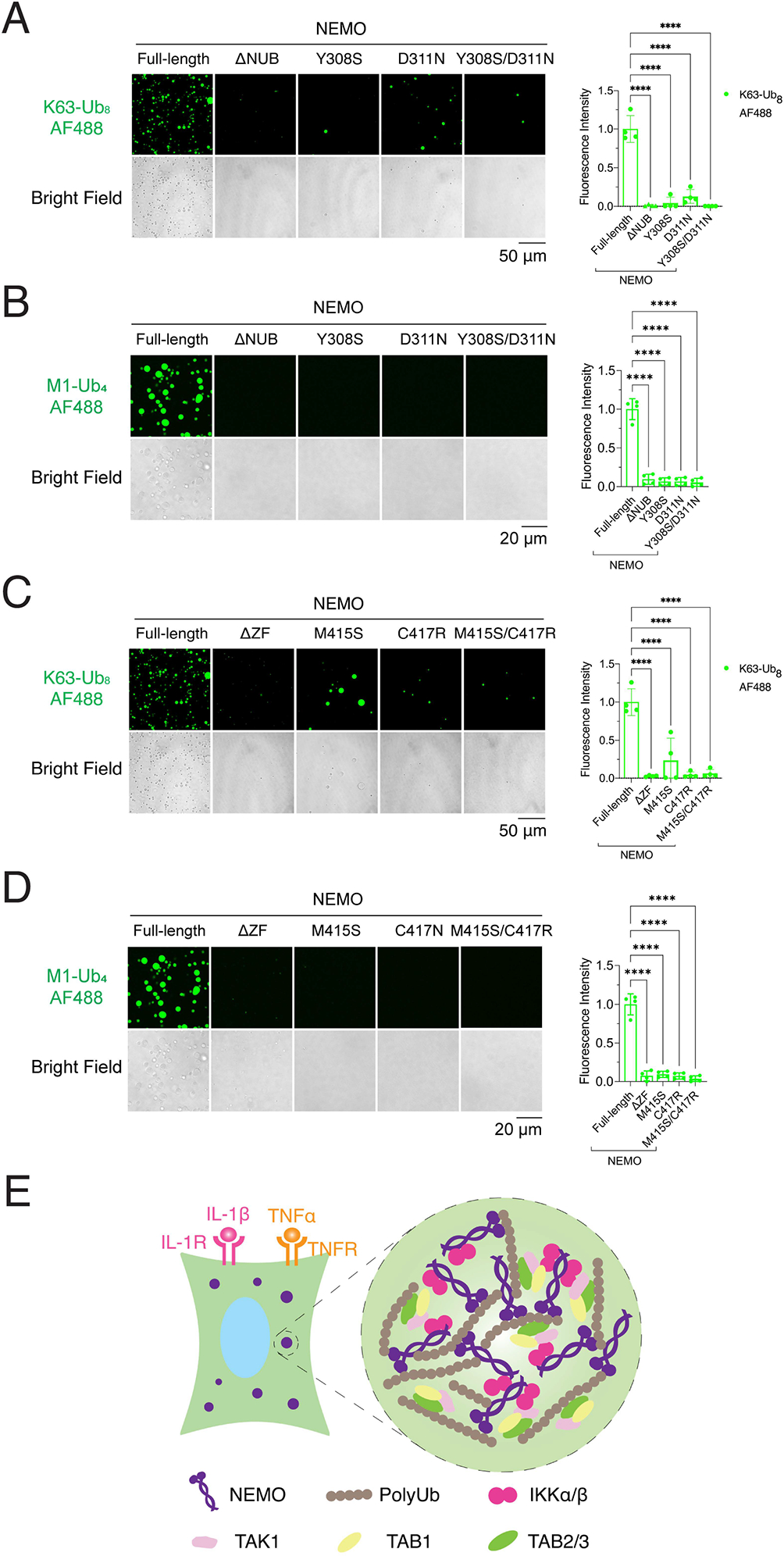
NEMO mutants linked to human diseases are defective in phase separation with polyubiquitin chains (A) and (C) Left panel: representative images of phase separation by incubating K63-Ub_8_ with NEMO or mutants as indicated. Right panel: quantification of fluorescence intensity of liquid droplets. Shown are means ± SD. n = 4 areas. These images are from the same experiment as in Figure 5D. (B) and (D) Left panel: representative images of phase separation by incubating M1-Ub_4_ with NEMO or mutants as indicated. Right panel: quantification of fluorescence intensity of liquid droplets. Shown are means ± SD. n = 4 areas. These images are from the same experiment as in Figure 5E. (E) Schematic of NEMO–PolyUb interactions that drive their liquid phase separation. Stimulation of cells with TNFα or IL-1β leads to the formation of K63-linked and linear polyUb chains that bind to NEMO/IKK and TAB2/TAK1 complex, driving the formation of liquid droplets in which the TAK1 and IKK complexes are condensed and activated. In (A), (B), (C), and (D): One-way analysis of variance (ANOVA); ****, P < 0.0001. See also Figure S15 and S16.

NEMO mutations have been found in patients with X-linked ectodermal dysplasia with immunodeficiency (EDA-ID) (Maubach et al., 2017). Several of these mutations occur in the ubiquitin binding domains. Specifically, the disease-causing mutations D311N and C417R are in the NUB and ZF domains, respectively (Doffinger et al., 2001; Smahi et al., 2000). These mutations, as well as the Y308S (in the NUB domain) and M415S (in the ZF domain) mutations have been found to impair polyUb binding and IKK activation (Cordier et al., 2009; Ea *et al.*, 2006; Wu *et al.*, 2006) (Figure S15). All of these ubiquitin-binding mutants of NEMO formed fewer liquid droplets with K63-Ub_8_, M1-Ub_4_, or with enzymatically synthesized polyUb than WT NEMO (Figure 7A–D and S16), consistent with the impaired ability of mutant NEMO to activate IKK (Cordier *et al.*, 2009; Doffinger *et al.*, 2001; Ea *et al.*, 2006). These results indicate that disease-associated mutations of NEMO impair its ability to bind polyUb and form liquid condensates, leading to defective NF-kB activation and therefore resulting in immunodeficiency.

## DISCUSSION

IKK was first identified as a 700-kDa kinase complex that was activated by polyUb chains (Chen et al., 1996). Subsequently, several proteins including TAB2 and NEMO were found to bind K63-linked and linear polyUb chains (Ea *et al.*, 2006; Kanayama *et al.*, 2004; Rahighi *et al.*, 2009; Wu *et al.*, 2006), but the mechanism by which polyUb activates IKK has remained elusive. Here we show that polyUb binding to NEMO induces a robust phase transition to liquid-like droplets, which function as a microreactor that concentrates both enzymes and substrates, greatly facilitating activation of TAK1 and IKK and the reactions they catalyze (Figure 7E). This physicochemical mechanism provides the mechanistic basis for why NEMO and its binding to polyUb are essential for IKK activation. PolyUb-induced NEMO phase separation also provides a mechanism for a switch-like response to inflammatory cytokines such as IL-1β and TNFα; this is made possible by the multivalent interactions between the ubiquitin binding domains (NUB and ZF) of NEMO and polyUb in a manner that depends on the length of the polyUb chains. The activation of E3 ubiquitin ligases such as TRAF6 by upstream signals ensures synthesis of long polyUb chains which not only facilitate NEMO phase separation but also counteract the degradation of these chains by cellular deubiquitinating enzymes (DUBs). These DUBs not only prevent the spurious activation of NF-κB in unstimulated cells, but also help to terminate inflammatory signaling by facilitating the clearance of NEMO condensates through degradation of polyUb within the condensates. Interestingly, a mouse NEMO K270A mutant was reported to be constitutively active through enhanced self-association of NEMO CoZi domains (mouse aa250–338) (Bloor et al., 2008). This mutation may enhance phase separation of NEMO in the absence of polyUb binding. Consistent with this model, NEMO K270A mutation was recently reported to form puncta in cells in the absence of stimulation and the mice bearing this mutation developed inflammatory diseases (Adapala et al., 2020).

While our data clearly demonstrated that NEMO binding to polyUb leads to the formation of the NEMO condensates in which IKK is activated, the role of polyUb in the NF-κB pathway is not limited to its ability to bind NEMO. Indeed, other proteins such as TAB2 also bind K63-linked polyUb and it has been shown that this binding is essential for the activation of TAK1 and IKK (Kanayama *et al.*, 2004; Xia *et al.*, 2009). It’s likely that polyUb binding to both TAB2 and NEMO, which in turn recruit TAK1 and IKKs, respectively, leads to the formation of liquid droplets that contain both TAK1 and IKK complexes, thereby greatly facilitating IKK phosphorylation by TAK1, resulting in IKK activation. Further characterization of the dynamics and compositions of the NEMO condensates should provide deeper insights into the mechanism of NF-κB activation. The ability of polyubiquitin chains to drive phase separation through multivalent interactions may underlie its signaling functions in diverse cellular processes especially those that are distinct from its traditional function of targeting protein degradation by the proteasome (Chen and Sun, 2009; Dao and Castaneda, 2020; Sun et al., 2018; Yasuda et al., 2020; Zaffagnini et al., 2018). The mechanism by which polyubiquitin chains activate the NEMO/IKK complex is reminiscent of how DNA activates the DNA-sensing enzyme cGAS (Du and Chen, 2018) and how double-stranded RNA activates the NLRP6 inflammasome (Shen et al., 2021): all relies on multivalent weak interactions to drive liquid phase separation, which generates a switch-like response to stimulation.

### Limitations of the study

While our study has demonstrated that polyUb binding to NEMO leads to the formation of liquid condensates containing the IKK complex that is then activated, the molecular compositions of the condensates formed within the stimulated cells need to be further characterized. Data presented in this paper showed that the NEMO condensates contained TRAF6 and the TAK1 kinase complex, but how the TRAF6 ubiquitin E3 ligase and TAK1 kinase are activated by upstream signals remain to be investigated. A potential role of deubiquitination enzymes in dissolving the NEMO condensates in cells as a feedback mechanism to turn off IKK activation also requires further studies. Future research should also investigate whether and how K63 and linear polyubiquitination may cooperate to enhance IKK activation. In addition, new tools that enable specific and precise interrogation of phase separation without interfering with other properties of NEMO should help dissect the specific role of NEMO phase separation in NF-κB signaling.

## STAR METHODS

Detailed methods are provided in the online version of this paper and include the following:

### RESOURCE AVAILABILITY

#### Lead contact

Further information and requests for resources and reagents should be directed to and will be fulfilled by the Lead Contact, Zhijian James Chen (zhijian.chen@utsouthwestern.edu).

#### Materials availability

All unique reagents generated in this study are available from the lead contact upon request.

#### Data and code availability

- Original western blot images and microscopy data reported in this paper will be shared by the lead contact upon reasonable request.
- This paper does not report original code.
- Any additional information required to reanalyze the data reported in this paper is available from the lead contact upon reasonable request.

### EXPERIMENTAL MODEL AND SUBJECT DETAILS

#### Mammalian cell culture

U2OS (ATCC® HTB-96™, female), HCT116 (ATCC® CCL-247™, male) and BJ-5ta (ATCC® CRL-4001™, male) cells were grown according to methods provided by ATCC (American Type Culture Collection). Stable cell lines derived from these cell lines and experimental treatments are described in Method Details.

##### *Escherichia coli* strains

BL21/pLys *E. coli* strains were grown at 37 °C, 230 rpm in LB media. For expression of recombinant proteins, they were transformed by expression plasmids (see below), propagated at 37 °C in TB (Terrific broth) media, and induced with IPTG at 18 °C.

### METHOD DETAILS

#### Cell Lines Construction

U2OS^NEMO KO^ cell line was generated by using CRISPR/Cas9 technique. Specifically, U2OS cells were infected with lentiviruses harboring Cas9, sgRNA and a puromycin-resistance gene. After puromycin (1 μg/ml) selection, single cell clones with confirmed NEMO deletion were selected for studies.

HCT116^NEMO KO^ cells were generated by knocking out NEMO using the same CRIPSR/Cas9 technique.

To establish HCT116^NEMO KO^-GFP-TRAF6 cell line, HCT116^NEMO KO^ cells were infected with lentiviruses expressing GFP-TRAF6 and a blasticidin-resistance gene. After blasticidin (10 μg/ml) selection, surviving cells were selected for studies.

To generate HCT116^NEMO KO^-GFP-TRAF6/mCherry-NEMO [Full-length, ΔNUB (Δ292–322), ΔZF (Δ389–419) or ΔNUB-ZF (Δ292–419)] cell lines, HCT116^NEMO KO^-GFP-TRAF6 cells were infected with lentiviruses expressing a full-length or truncated mCherry-NEMO and a neomycin-resistance gene. After neomycin (400 μg/ml) selection, surviving cells were sorted on a cell sorter (MoFlo XDP, Beckman Coulter) based on NEMO expression levels (mCherry intensity). Cells expressing mCherry-NEMO or its mutants at levels comparable to that of endogenous NEMO were selected for studies.

To generate a U2OS^mCherry-NEMO knock-in^ cell line, U2OS cells were transfected with a lentiCRISPRv1 plasmid for expressing Cas9 and sgRNA targeting the translation start site of NEMO gene locus, and with a donor DNA template carrying mCherry gene flanked by ~ 800-bp homology arms complementary to the target site of the NEMO gene locus. 7 days after transfection, cells were sorted on a cell sorter (FACSAria II, BD) and those expressing mCherry were harvested (0.1% of total cells sorted) and selected for single clones. The single clones were verified by western blotting of NEMO and sanger sequencing of NEMO gene locus, and the ones with both alleles encoding mCherry fused in-frame with NEMO were selected for further studies.

#### Protein Expression, Purification and Labeling

Human recombinant NEMO (FL-h-NEMO) and mutants, including ΔNUB (Δ292–322), ΔZF (Δ389–419), ΔNUB-ZF (Δ292–419), Y308S, D311N, Y308S/D311N, M415S, C417R, and M415S/C417R were expressed in the *E.coli* strain BL21/pLys harboring a pGEX-4T1 plasmid encoding indicated GST-NEMO protein. After induction with 0.5 mM IPTG at 18 °C for 20 hours, bacteria were collected by centrifugation and lysed by sonication in a buffer containing 20 mM Tris-HCl, pH 8.0, 300 mM NaCl, and 0.2 mM PMSF. After centrifugation, clear lysate supernatant was incubated with Glutathione Sepharose 4B resin (GE Healthcare), loaded onto gravity flow columns, washed with lysis buffer and the GST tag was cleaved on column with Thrombin (Millipore, 69671) at 4 °C overnight. Cleaved protein was collected, concentrated, and dialyzed against a buffer containing 20 mM Tris-HCl, pH 7.5 and 150 mM NaCl. Recombinant NEMO protein was labeled with either Alexa Fluor 555 or Alexa Fluor 647 using Alexa Fluor™ Protein Labeling Kit (ThermoFisher). The labeling stoichiometry was estimated at 2 moles of fluorescent dye per mole of NEMO for each dye.

K63-Ub_8_ or M1-Ub_4_ was labeled with Alexa Fluor 488 using Alexa Fluor™ 488 Microscale Protein Labeling Kit (Cat. A30006, ThermoFisher) and the labeling stoichiometry was 2 moles of Alexa Fluor 488 per mole of K63-Ub_8_ or M1-Ub_4_.

His_6_-tagged E1 was expressed in Sf9 cells and purified using Ni-NTA agarose beads (QIAGEN). His_6_-tagged UBC13 and mCherry-TRAF6 (aa1–358) were expressed in BL21/pLys and affinity purified. GST-tagged UEV1A, IL-1β (matured form), and TNFα were similarly expressed in BL21/pLys and affinity purified using Glutathione Sepharose 4B resin (GE Healthcare).

The TAK1 kinase complex (containing TAK1, TAB1 and TAB2) was purified from a HEK293T cell line stably expressing Flag tagged TAB2 using anti-Flag (M2) agarose beads.

To purify endogenous IKK complex lacking NEMO, S100 from a NEMO deficient murine pre-B cell line (1.3E2) was loaded onto a HiTRAP Q HP column, and fractions containing IKKα/β were eluted with 200–300 mM NaCl. After precipitation with ammonium sulphate (16–30%) and dialysis, proteins were loaded onto a Superdex-200 gel filtration column. Fractions containing IKKα/β were incubated with NEMO proteins and tested for IKK activity.

#### In vitro Phase Separation Assay

To test phase separation of NEMO with polyUb of defined linkages and lengths, recombinant NEMO protein (3% Alexa Fluor 555-labeled) was mixed with polyUb (3% Alexa Flour 488-labeled) in a buffer containing 20 mM Tris-HCl, pH 7.5, 150 mM NaCl, 5 mM ATP, 2 mM DTT and 0.2 mg/ml BSA. Mixtures were incubated in 96-well plates (Corning™ 4580) coated with 30 mg/ml BSA (Sigma) at 37 °C and images were captured at indicated times with a Nikon A1R+ confocal microscope equipped with a 40× oil objective, a Nikon A1 camera, and a X-Cite 120 LED illuminator at 37 °C. Fluorescent intensities were measured by quantifying the total fluorescence of areas using ImageJ (Open source Java program from NIH).

To test phase separation of NEMO with enzymatically synthesized polyUb, recombinant NEMO protein (3% Alexa Fluor 647-labeled) was mixed with 14.5 μM ubiquitin (3% Fluorescein-labeled), 85 nM E1, 333 nM E2 (UBC13/UEV1A), in the presence or absence of 156 nM E3 (mCherry-TRAF6[aa1–358]) in a buffer containing 20 mM Tris-HCl, pH 7.5, 5 mM MgCl_2_, 5 mM ATP, 2 mM DTT and 0.2 mg/ml BSA. Mixtures were incubated at 37 °C for an hour in 96-well plates (Corning™ 4580) coated with 30 mg/ml BSA (Sigma) and then images were captured as above.

#### In vitro IKK activity assay

To measure IKKα/β activation by NEMO and K63-Ub_8_, the purified IKK complex from 1.3E2 cells were incubated with purified TAK1 complex, 1 μM K63-Ub_8_ and NEMO at indicated concentrations in a reaction mixture (10 μl) containing GST-IκBα (amino acids 1–62, 0.4 μg), 20 mM Tris-HCl, pH 7.5, 150 mM NaCl, 2 mM ATP, 2 mM MgCl_2_, 2 mM DTT, 0.2 mg/ml BSA. After incubation at 30 °C for 1 h, IKK activity was analyzed by immunoblotting using an antibody against phospho-IKKα/β or phospho-IκBα.

#### Cellular FRAP Assays

Cellular fluorescence recovery after photobleaching (FRAP) experiments were performed at 37 °C in a live-cell-imaging chamber. HCT116^NEMO KO^-GFP-TRAF6/mCherry-NEMO cells were grown on chambered cover glass until they reached the desired density. Cells were then stimulated with IL-1β (1 μg/ml) for 15 min. NEMO-TRAF6 condensates were fully photobleached by using 488-nm and 561-nm lasers on a Nikon A1R+ confocal microscope equipped with a 40× oil objective, a Nikon A1 camera, and a X-Cite 120LED illuminator at 37 °C. Time-lapse images were acquired over a 70-second time course after bleaching with 3.89-second interval. Images were processed by ImageJ. Fluorescence intensities of regions of interest (ROIs) were corrected by unbleached control regions and then normalized to pre-bleached intensities of the ROIs. The corrected and normalized data were fit to the single exponential model by GraphPad Prism 8:

$$I_{t}=I_{0}+\left(I_{\infty }-I_{0}\right)\left(1-e^{-kt}\right)$$

where I_0_ was the intensity at the start of recovery after bleaching, I_∞_ was the plateau intensity, and k was the exponential constant. τ was calculated by the reciprocal of k. The recovery rate was calculated by I_∞_ divided by the fluorescence intensity before bleaching. t_1/2_ represents the time point achieving half maximal recovery intensity (I_∞_−I_0_)/2.

For HCT116^NEMO KO^-GFP-TRAF6/mCherry-NEMO cells stimulated with TNFα (1 μg/ml) for 10 min, NEMO condensates were fully photobleached by using 561-nm laser at 37 °C on a Andor spinning disk confocal microscope system equipped with Nikon Ti motorized microscope (a 60× oil objective), CSUX1 Spinning Disk Confocal head (Yokogawa), an Andor iXon EMCCD camera and a Neo sCMOS camera. Time-lapse images were acquired over a 120-second time course after bleaching with 5-second interval. Images were processed by ImageJ and fluorescence recovery was analyzed by GraphPad Prism 8 as described above.

#### Immunofluorescence Staining

BJ-5ta, U2OS, or U2OS^mCherry-NEMO knock-in^ cells were seeded on 12-mm circular coverslips at 10^5^ cells/well in a 12-well tissue-culture plate overnight and then treated with IL-1β (1 μg/ml) for 10 min or TNFα (1 μg/ml) for 5 min. Cells were washed with PBS and permeabilized by 0.02% Saponin in PBS for 3 min, except for staining K63-linked polyubiquitin chains, in which case cells were permeabilized by 0.02% Saponin in PBS supplemented with 10 mM N-ethylmaleimide for 10 min. Cells were then fixed with 4% paraformaldehyde in PBS at room temperature for 15 min. Fixed cells were blocked by 5% BSA in PBS for 1 hour followed by incubation with indicated antibodies in PBS containing 5% BSA at 4°C overnight. After 3 times of 5-min washes with PBS, cells were incubated with Alexa Flour 488 or Alexa Flour 555 conjugated goat-anti-rabbit or goat-anti-mouse IgG secondary antibodies for 1 hour at room temperature. After 3 times of 5-min washes with PBS, the coverslips were mounted in Vectashield® mounting medium with DAPI (Vector Laboratories, H-1200). Cells were then examined under a Nikon CSU-W1 SoRa Spinning disk confocal microscope equipped with a 60× oil objective and a Hamamatsu Orca-Fusion sCMOS camera. Signals of DAPI, Alexa Flour 488, Alexa Flour 555 (or mCherry) were examined by 405, 488, 561 nm laser lines respectively. To improve the NEMO puncta signal above the background, we used 0.02% Saponin to permeabilize cell membrane to allow free NEMO but not NEMO puncta to leak out before cells were fixed with 4% paraformaldehyde. Images were analyzed by ImageJ and the NEMO puncta formed in cells by IL-1β or TNFα stimulation were quantified by counting the number of puncta within 0.3–1 μm and using the cell counter module of ImageJ. To quantify the puncta (i.e. K63-polyUb puncta) that co-localized with NEMO punctum, the distances between the centers of K63-polyUb and NEMO puncta that were less than 0.2 μm were determined as true co-localization. The images of different fluorescent channels were captured at the same z-slice and the same z-slice was used for testing NEMO puncta colocalization with K63-polyUb puncta. Cells that contained NEMO puncta after IL-1β or TNFα treatment represented > 90% of stimulated cells.

#### Subcellular Fractionation and IKK Activity Assay

##### Subcellular fractionation

BJ-5ta or U2OS cells were stimulated with IL-1β (1 μg/ml) for 10 min or TNFα (1 μg/ml) for 5 min before cells (5 × 10^7^) were lysed by passing through a 27G1/2 needle six times in 500 μl hypotonic buffer (10 mM Tris-HCl, pH 7.5, 5 mM KCl, and 3 mM MgCl_2_) supplemented with 1 mM sodium orthovanadate, 0.5 mM DTT, and a protease inhibitor cocktail. The homogenate was centrifuged at 1,000 × g for 10 minutes and the pellet (P1) was washed with hypotonic buffer and resuspended in a desired buffer for analysis. The supernatant (S1) was further centrifuged at 20,000 × g for 10 minutes and the pellet (P20) was washed with hypotonic buffer and resuspended into a desired buffer for analysis. The supernatant was collected as S20 for analysis or further fractionated by centrifugation at 100,000 × g for 1 hour or by Optiprep density gradient ultracentrifugation (see below). The supernatant and pellet after 100,000 × g were designated as S100 and P100, respectively. The P100 was resuspended with the same lysis buffer (half of the S100 volume) by briefly pipetting up and down the pellet, letting the tube sit on ice for 1~2 hours followed by resuspending the pellet by pipetting.

##### Optiprep gradient ultracentrifugation

Optiprep solutions at different densities (5%, 10%, 15%, 20%, 25%, and 30% in Iso-osmotic buffer) were prepared and 200 μl of each solution was layered on top of each other from higher density (bottom) to lower density (top) in a centrifuge tube. Then, 100 μl of the S20 fraction was loaded on top of the layered gradient and the tubes were subjected to ultracentrifugation at 100,000 × g for 2 h. After ultracentrifugation, 100 μl of each fraction was collected by pipetting carefully from top to bottom (13 fractions).

##### IKK activity assay

To measure the activity of IKK in each subcellular fraction, 20 μl of each fraction was incubated with GST-IκBα (amino acids 1–62, 0.4 μg) in a reaction mixture (30 μl) containing 20 mM Tris-HCl, pH 7.5, 5 mM ATP, 5 mM MgCl_2_, 2 mM DTT, 0.2 mg/ml BSA. After incubation at 30 °C for 1 h, IKK activity was analyzed by immunoblotting using an antibody against phospho-IκBα.

##### Imaging NEMO condensates in subcellular fractions

U2OS^mCherry-NEMO knock-in^ cells were grown on 15-cm dishes to an 80–90% density. Cells were stimulated with IL-1β (1 μg/ml) for 10 min or TNFα (1 μg/ml) for 5 min before cells (5 15-cm dishes, ~5 × 10^7^ cells) were permeabilized by 0.02% Saponin in PBS supplemented with 10 mM N-ethylmaleimide, 5 mM sodium orthovanadate and a protease inhibitor cocktail for 5 min. Cells were then washed with hypotonic buffer (10 mM Tris-HCl, pH 7.5, 5 mM KCl, and 3 mM MgCl_2_) once, and scraped into hypotonic buffer supplemented with 5 mM sodium orthovanadate, and a protease inhibitor cocktail. Cells were further lysed by passing through a 27G1/2 needle six times. The homogenate was fractionated by differential centrifugation as described above except that the volume of P100 resuspension was 1/10 of the S100 volume. Fractions of S20, S100 and P100 were directly examined under a Nikon CSU-W1 SoRa Spinning disk confocal microscope equipped with a 60× oil objective and a Hamamatsu Orca-Fusion sCMOS camera. mCherry-NEMO condensates were imaged by 561 nm laser line. Images were analyzed by ImageJ and NEMO condensates were counted using the cell counter module of ImageJ.

##### Estimating the percentage of catalytically active fraction of the NEMO/IKK complex

NEMO protein level in P100 was about half of that in S100 (Figure 4F and S10C), and the volume of P100 was half of that of S100, so NEMO protein in P100 was ~20% of total NEMO protein in cells. Since S100 had barely detectable IKK activity, we estimated that ~20% of total NEMO protein was catalytically active, which was enriched in P100.

#### Immunoblotting

Cell lysates were boiled in 1× Laemmli sample buffer for 5 min, and then electrophoresed on 4–20% Tris-Glycine eXtended (TGX) precast protein gels (Biorad). Proteins were transferred to 0.2 μm polyvinylidene difluoride (PVDF) membranes by using a Trans-Blot Turbo Transfer System (Biorad). The membranes were blocked for 30 min in 5% non-fat milk, and then incubated for 2 hours at room temperature or overnight at 4 °C with a primary antibody. After 3 times of 5-min wash with TBST, the membranes were immunoblotted with alkaline phosphatase (AP) conjugated goat-anti-rabbit IgG or goat-anti-mouse IgG secondary antibody (Promega) for 1 hour at room temperature. After 3 times of 5-min wash with TBST, blots were developed using 5-Bromo-4-chloro-3-indolyl phosphate (BCIP, ThermoFisher) and Nitro blue tetrazolium (NBT, ThermoFisher) as substrates. The blots were then imaged on a ChemiDoc Imaging System (Biorad) and the bands on blots were quantified by ImageJ (Open source Java program from NIH) or the immunoblotting quantification software Image Studio™ Lite (LI-COR Biosciences).

#### Live Cell Imaging

Cells were grown on chambered cover glasses to 70–80% confluency, at which time cells were stimulated with IL-1β (1 μg/ml) or TNFα (1 μg/ml). Live cell images were immediately captured every 10 seconds for 1 hour (IL-1β stimulation) or 30 min (TNFα stimulation) under an Andor spinning disk confocal microscope system equipped with a Nikon Ti motorized microscope (with 60× oil objective), a CSUX1 Spinning Disk Confocal head (Yokogawa), an Andor iXon EMCCD camera and a Neo sCMOS camera. Images were analyzed by ImageJ. To quantify the number of cellular condensates, backgrounds of images were first subtracted by using the module of subtract background on ImageJ, and then condensates (~ 0.3–1 μm in diameter) in cells were spotted and counted using the plugin of SpotCounter on ImageJ (the threshold of fluorescent intensity in detecting NEMO puncta was set above that of free NEMO). For tracking the fusion of two condensates as shown in Figure 2E–F, trajectories and positions of condensates were obtained using the plugin of TrackMate on ImageJ. Cells that contained NEMO puncta after IL-1β or TNFα treatment represented > 90% of stimulated cells.

#### Protein Sequence Alignment

The sequences of NEMO proteins across different species were obtained from National Center for Biotechnology Information (NCBI). Sequence alignment was performed using Clustal Omega from the European Molecular Biology Laboratory’s European Bioinformatics Institute (EMBL-EBI). Results of the alignment were illustrated using the ESPript3.0 software.

### QUANTIFICATION AND STATISTICAL ANALYSIS

The statistical tests and numbers of independent replicates per experiment are indicated in the figure legends.

## Supplementary Material

Movie S1 Live cell imaging video of the phase condensation of NEMO and NEMO-mutants in response to IL-1β stimulation, related to Figure 6 HCT116^NEMO KO^-GFP-TRAF6 cells reconstituted with mCherry-tagged NEMO or indicated mutants were stimulated with IL-1β, followed by live cell imaging.

Movie S2 Live cell imaging video of the phase condensation of NEMO and NEMO mutants in response to TNFα stimulation, related to Figure 6 HCT116^NEMO KO^-GFP-TRAF6 cells reconstituted with mCherry-tagged NEMO or indicated mutants were stimulated with TNFα, followed by live cell imaging

Supplementary Material

## Figures and Tables

**KEY RESOURCES TABLE:** 

REAGENT or RESOURCE	SOURCE	IDENTIFIER
**Antibodies**		
Mouse monoclonal anti-IκBα	Cell Signaling Technology	Cat# 4814
Mouse monoclonal anti-phospho-IκBα (Ser32/36)	Cell Signaling Technology	Cat# 9246
Mouse monoclonal anti-Ubiquitin	Cell Signaling Technology	Cat# 3936
Mouse monoclonal anti-IKKα	Cell Signaling Technology	Cat# 11930
Rabbit monoclonal anti-mCherry	Cell Signaling Technology	Cat# 43590
Rabbit monoclonal anti-phospho-TAK1 (Thr187)	Cell Signaling Technology	Cat# 4536
Rabbit monoclonal anti-TAK1 (Western Blotting)	Cell Signaling Technology	Cat# 5206
Rabbit monoclonal anti-TAB2	Cell Signaling Technology	Cat# 3745
Rabbit monoclonal anti-H2A	Cell Signaling Technology	Cat# 2578
Rabbit monoclonal anti-GAPDH	Cell Signaling Technology	Cat# 5174
Rabbit monoclonal anti-A20	Cell Signaling Technology	Cat# 5630
Rabbit monoclonal anti-CYLD	Cell Signaling Technology	Cat# 8462
Rabbit monoclonal anti-Cyclophilin B	Cell Signaling Technology	Cat# 43603
Rabbit monoclonal anti-phospho-IKKα/β (Ser176/180)	Cell Signaling Technology	Cat# 2697
Rabbit polyclonal anti-TRAF6	Santa Cruz biotechnology	Cat# sc-7221
Mouse monoclonal anti-IRAK1	Santa Cruz biotechnology	Cat# sc-5288
Mouse monoclonal anti-IKKγ	BD Biosciences	Cat# 611306
Rabbit monoclonal anti-TRAF2	Abcam	Cat# ab126758
Rabbit monoclonal anti-TAK1 (Immunostaining)	Abcam	Cat# ab109526
Rabbit polyclonal anti-RNF31/HOIP	Abcam	Cat# ab187976
Rabbit polyclonal anti-cIAP1	ProteinTech Group	Cat# 10022-1-AP
Mouse monoclonal anti-K63-linkage-polyubiquitin	Thermo Fisher Scientific	Cat# 14-6077-82
Goat anti-Rabbit Secondary Antibody Alexa Fluor 488	Thermo Fisher Scientific	Cat# A32731
Goat anti-Rabbit Secondary Antibody Alexa Fluor 555	Thermo Fisher Scientific	Cat# A32732
Goat anti-Mouse Secondary Antibody Alexa Fluor 488	Thermo Fisher Scientific	Cat# A32723
Goat anti-Mouse Secondary Antibody Alexa Fluor 555	Thermo Fisher Scientific	Cat# A32727
Mouse monoclonal anti-α-Tubulin	Millipore Sigma	Cat# T6199
**Chemicals, peptides, and recombinant proteins**		
K48-Ub_2_	R&D Systems	Cat# UC-200B
K63-Ub_2_	R&D Systems	Cat# UC-300B
M1-Ub_2_	R&D Systems	Cat# UC-700B
K6-Ub_4_	R&D Systems	Cat# UC-15
K11-Ub_4_	R&D Systems	Cat# UC-45
K29-Ub_4_	R&D Systems	Cat# UC-83
K33-Ub_4_	R&D Systems	Cat# UC-103
K48-Ub_4_	R&D Systems	Cat# UC-210B
K63-Ub_4_	R&D Systems	Cat# UC-310B
M1-Ub_4_	R&D Systems	Cat# UC-710B
K63-Ub_3_	R&D Systems	Cat# UC-315B
K63-Ub_8_	R&D Systems	Cat# UC-318B
K63-Ub_2-7_	R&D Systems	Cat# UC-330
K48-Ub_2-7_	R&D Systems	Cat# UC-230
K63-Ub_2-12_	UBPBio	Cat# D2600
M1-Ub_2-7_	Enzo Life Sciences	Cat# BML-UW1010-0100
Linear polyubiquitin chains	Enzo Life Sciences	Cat# BML-UW0825-0001
Recombinant human USP5 (Isopeptidase T)	R&D Systems	Cat# E-322
Recombinant human CYLD	R&D Systems	Cat# E-556
**Experimental models: Cell lines**		
U2OS	ATCC	Cat# HTB-96
HCT116	ATCC	Cat# CCL-247
BJ-5ta	ATCC	Cat# CRL-4001
**Software and algorithms**		
Prism 8	Graphpad	https://www.graphpad.com/
ImageJ	National Institutes of Health	https://imagej.nih.gov/ij/
Image Studio™ Lite	LI-COR Biosciences	https://www.licor.com/bio/image-studio-lite/
Clustal Omega	EMBL-EBI	https://www.ebi.ac.uk/Tools/msa/clustalo/
ESPript3.0	ESPript	https://espript.ibcp.fr/ESPript/ESPript/
