# Supplementary material for: Liquid Phase Separation of NEMO Induced by Polyubiquitin Chains Activates NF-κB
Source: Mol Cell. Author manuscript; Available in PMC 2022 Aug 25. (PMC9402427; doi:10.1016/j.molcel.2022.03.037)
Supplement: Supplementary Material [file NIHMS1829180-supplement-Supplementary_Material.pdf]

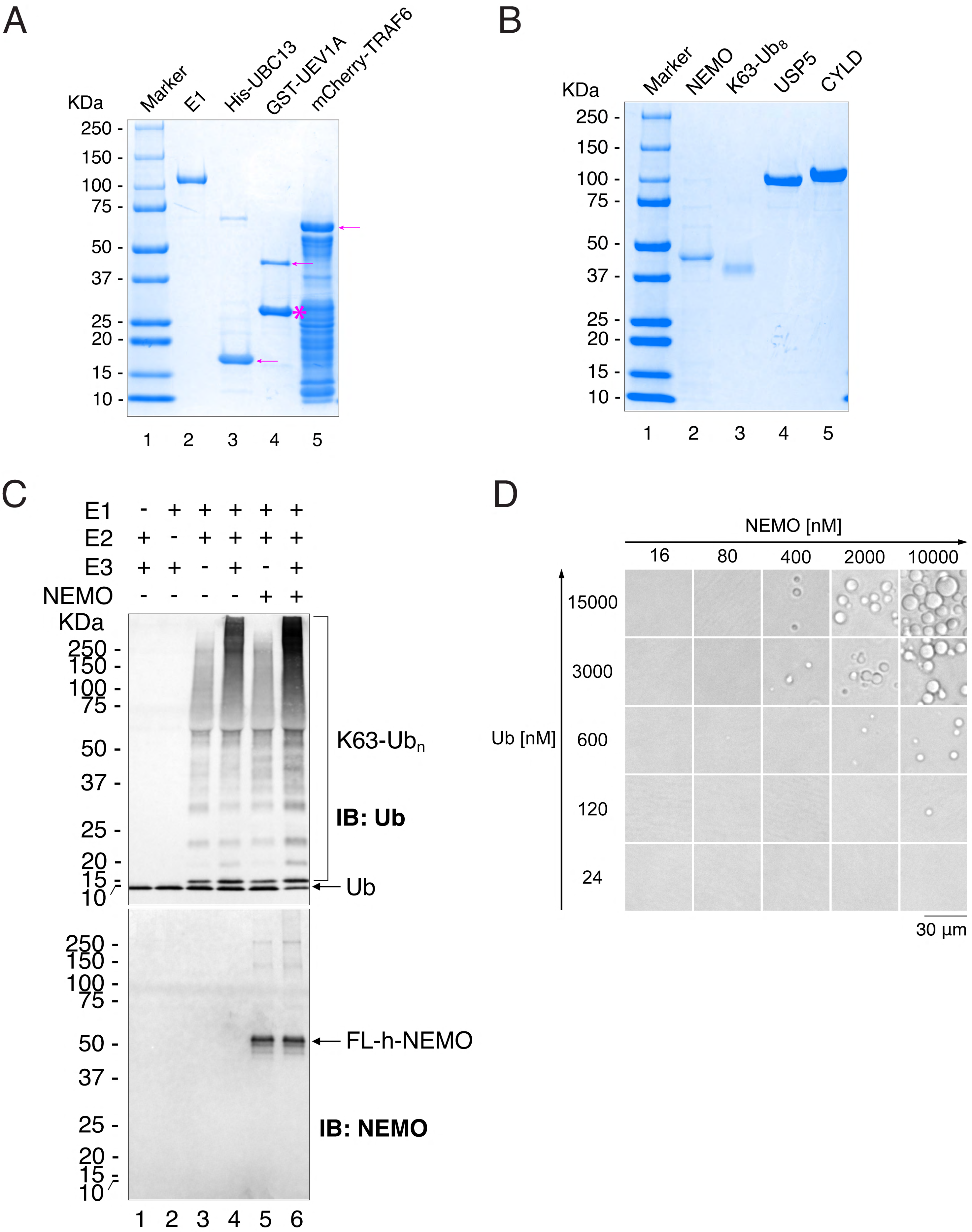
A
KDa
Marker
E1
His-UBC13
GST-UEV1A
mCherry-TRAF6
250 -
150 -
100 -
75 -
50 -
37 -
25 -
20 -
15 -
10 -
*
1 2 3 4 5
B
KDa
Marker
NEMO
K63-Ub8
USP5
CYLD
250 -
150 -
100 -
75 -
50 -
37 -
25 -
20 -
15 -
10 -
1 2 3 4 5
C
E1 - + + + + +
E2 + - + + + +
E3 + + - + - +
NEMO - - - - + +
KDa
250 -
150 -
100 -
75 -
50 -
37 -
25 -
20 -
15 -
10
K63-Ubn
IB: Ub
Ub
250 -
150 -
100 -
75 -
50 -
37 -
25 -
20 -
15 -
10
FL-h-NEMO
IB: NEMO
1 2 3 4 5 6
D
NEMO [nM]
16
80
400
2000
10000
Ub [nM]
15000
3000
600
120
24
30 µm

Figure S1. Polyubiquitin chains induce phase separation of NEMO, related to Figure 1

(A) Coomassie blue staining of purified recombinant E1 (118 KDa), E2 (His_6-UBC13, 17 KDa; GST-UEV1A, 43 KDa), and E3 (mCherry-TRAF6[aa1-358], 64 KDa). Arrows indicate the proteins of interest. The band marked by an asterisk is Glutathione S-transferase (GST). mCherry-TRAF6 was partially purified.

(B) Coomassie blue staining of purified recombinant proteins as indicated.

(C) Immunoblotting (IB) of enzymatically synthesized K63-polyUb and NEMO in the presence or absence of E1, E2 (UBC13/UEV1A), E3 (TRAF6) or NEMO as indicated. FL-h-NEMO: full-length human NEMO.

(D) Representative bright field images of NEMO liquid droplets induced by enzymatically synthesized K63-polyUb. Liquid droplets were imaged after mixing of human FL-NEMO with ubiquitin at indicated concentrations in a reaction containing E1, E2 (UBC13/UEV1A) and E3 (TRAF6). These images correspond to the phase separation diagram in Figure 1C.

A

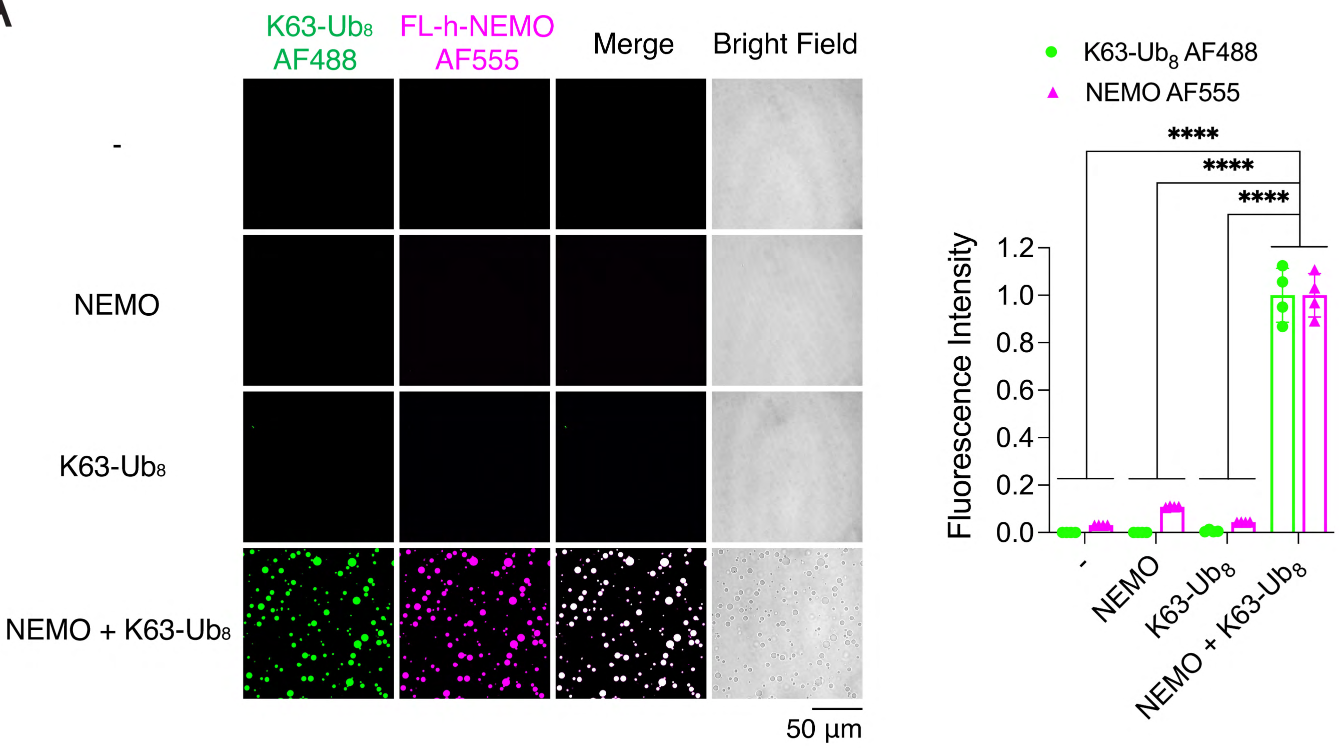

K63-Ub8 AF488
FL-h-NEMO AF555
Merge
Bright Field
-
NEMO
K63-Ub8
NEMO + K63-Ub8
50 µm
K63-Ub8 AF488
NEMO AF555
****
****
****
Fluorescence Intensity
1.2
1.0
0.8
0.6
0.4
0.2
0.0
-
NEMO
K63-Ub8
NEMO + K63-Ub8

B

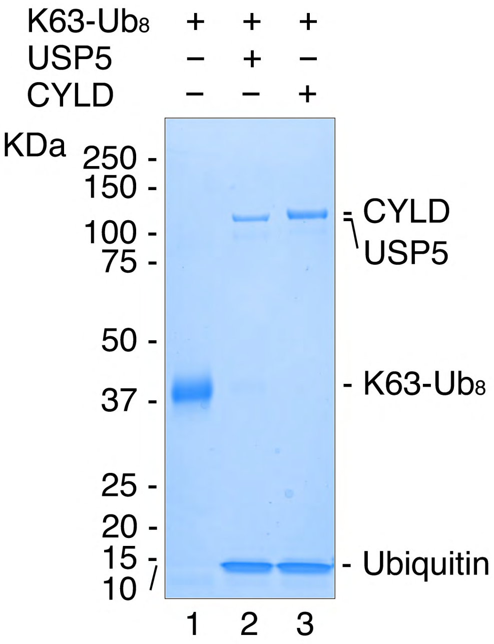

K63-Ub8 + + +
USP5 − + −
CYLD − − +
KDa
250
150
100
75
50
37
25
20
15
10
CYLD
USP5
K63-Ub8
Ubiquitin
1 2 3

Figure S2. K63-Ub_8 binding to NEMO induces their phase separation, related to Figure 1

(A) Left panel: representative fluorescent images of NEMO liquid droplets induced by K63-Ub_8 in the presence or absence of K63-Ub_8 or NEMO. Liquid droplets formed after mixing of 6 μM human FL-NEMO (3% was labeled with Alexa 555) with 2.25 μM K63-Ub_8 (3% was labeled with Alexa 488) for an hour at 37°C. Right panel: quantification of fluorescence intensity of liquid droplets. Shown are means ± SD. n = 4 areas. Two-way analysis of variance (ANOVA). ****P < 0.0001. These images are from the same experiment as in Figure 1D.

(B) Coomassie blue staining of K63-Ub_8 (theoretical molecular weight at 68 KDa) cleaved by USP5 (96 KDa) or CYLD (107 KDa).

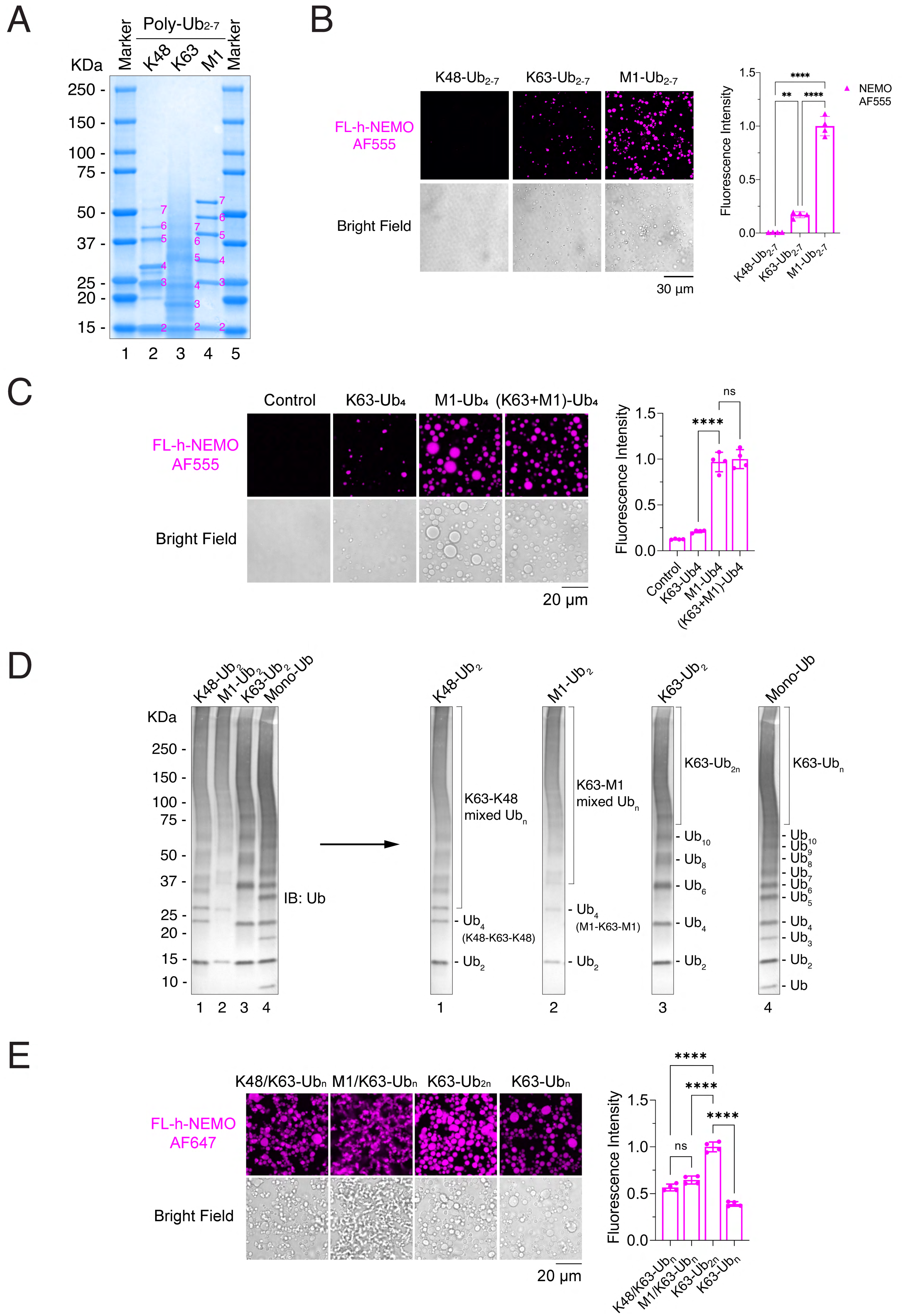

A
KDa
Marker
Poly-Ub2-7
K48
K63
M1
Marker
250 -
150 -
100 -
75 -
50 -
37 -
25 -
20 -
15 -
1 2 3 4 5
B
K48-Ub2-7
K63-Ub2-7
M1-Ub2-7
FL-h-NEMO AF555
Bright Field
30 μm
Fluorescence Intensity
NEMO AF555
C
Control
K63-Ub4
M1-Ub4
(K63+M1)-Ub4
FL-h-NEMO AF555
Bright Field
20 μm
Fluorescence Intensity
ns
D
K48-Ub2
M1-Ub2
K63-Ub2
Mono-Ub
KDa
IB: Ub
K63-K48 mixed Ubn
Ub4 (K48-K63-K48)
Ub2
K63-M1 mixed Ubn
Ub4 (M1-K63-M1)
K63-Ub2n
K63-Ubn
1 2 3 4
E
K48/K63-Ubn
M1/K63-Ubn
K63-Ub2n
K63-Ubn
FL-h-NEMO AF647
Bright Field
20 μm
Fluorescence Intensity
ns

Figure S3. NEMO phase separation is specifically induced by K63- and M1-linked polyUb, related to Figure 2

(A) Coomassie blue staining of Ub_{2-7} linked through K48, K63 or M1. Numbers (magenta color) indicate the bands of Ub_2 to Ub_7. K63-linked Ub_{2-7} appeared to have some minor smears that may represent impurity.

(B) Left panel: representative fluorescent images of NEMO liquid droplets induced by Ub_{2-7} linked through K48, K63, and M1. Right panel: quantification of fluorescence intensity of liquid droplets.

(C) Left panel: representative fluorescent images of NEMO liquid droplets induced by K63- (4.5 μM) or M1- Ub_4 (4.5 μM), or a mixture of both (2.25 μM + 2.25 μM). Liquid droplets formed after mixing of 6 μM human FL-NEMO (3% was labeled with Alexa 555) with 4.5 μM Ub_4 of different linkages for 1 hour at 37°C. Right panel: quantification of fluorescence intensity of liquid droplets.

(D) Immunoblotting (IB) of enzymatically synthesized hybrid K48/K63- and M1/K63-polyUb after mixing of 7.25 μM K48-Di-Ub, M1-Di-Ub, K63-Di-Ub or 14.5 μM ubiquitin in a reaction mixture containing E1, UBC13–UEV1A (E2), and TRAF6 (E3) for an hour at 37°C.

(E) Left panel: representative fluorescent images of NEMO liquid droplets induced by enzymatically synthesized hybrid K48/K63- or M1/K63- polyUb. Liquid droplets formed after mixing of 6 μM human FL-NEMO (3% was labeled with Alexa Fluor 555) with 7.25 μM K48-Di-Ub, M1-Di-Ub, K63-Di-Ub or 14.5 μM ubiquitin in a reaction mixture containing E1, UBC13–UEV1A (E2), and TRAF6 (E3) for an hour at 37°C. Right panel: quantification of fluorescence intensity of liquid droplets.

Data shown in (B), (C) and (E) are means ± SD. n = 4 areas. One-way analysis of variance (ANOVA). n.s., $P > 0.0332$; **$P < 0.0021$; ****$P < 0.0001$.

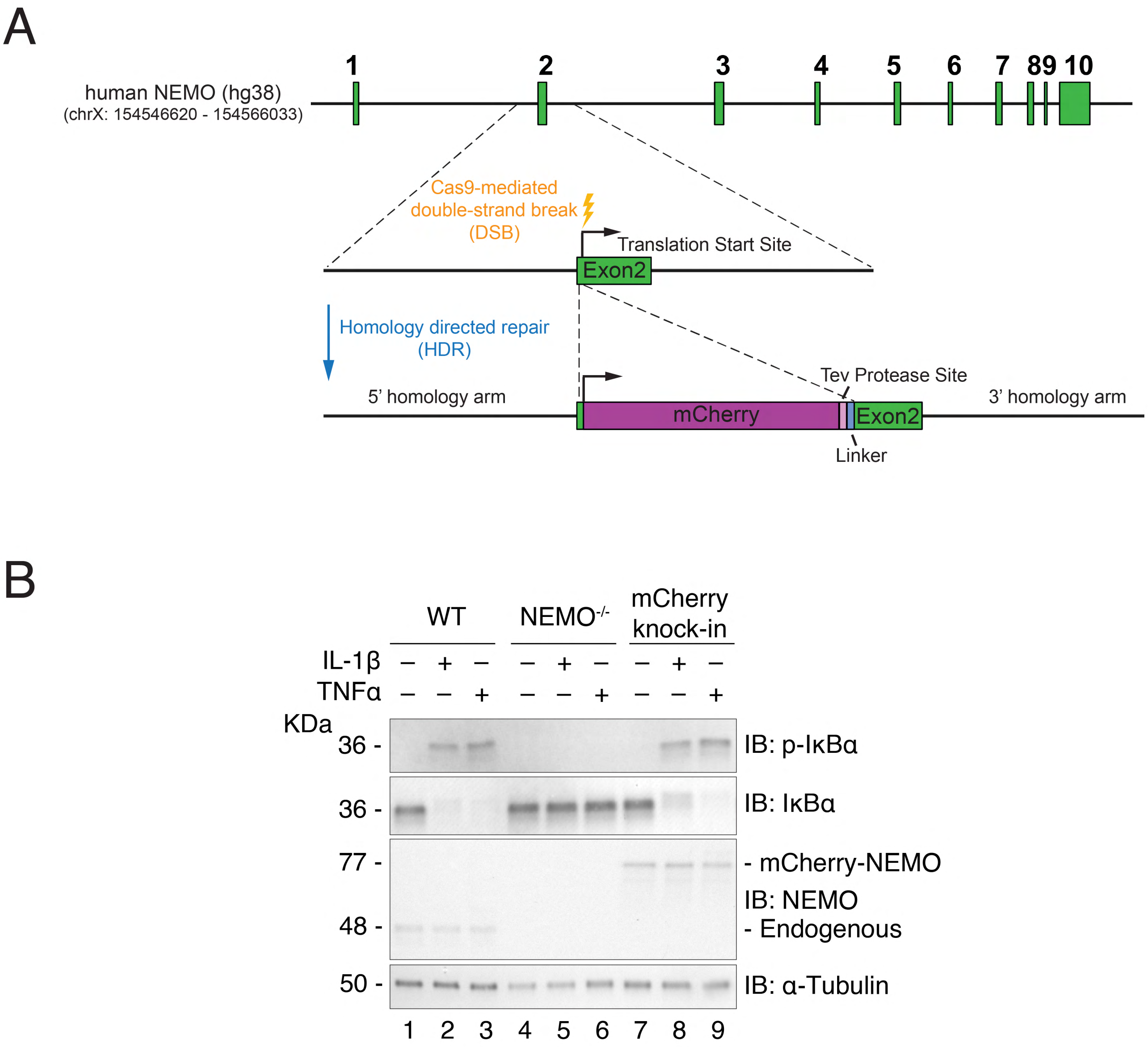
A
1
2
3
4
5
6
7
89
10
human NEMO (hg38)
(chrX: 154546620 - 154566033)
Cas9-mediated
double-strand break
(DSB)
Translation Start Site
Exon2
Homology directed repair
(HDR)
Tev Protease Site
5' homology arm
mCherry
Exon2
3' homology arm
Linker
B
WT
NEMO-/-
mCherry
knock-in
IL-1β
TNFα
KDa
36 -
IB: p-IκBα
36 -
IB: IκBα
77 -
- mCherry-NEMO
IB: NEMO
48 -
- Endogenous
50 -
IB: α-Tubulin
1 2 3 4 5 6 7 8 9

Figure S4. U2OS$^{mCherry\text{-}NEMO\ Knock\text{-}in}$ cells exhibited robust IκBα phosphorylation and degradation in response to stimulation with IL-1β or TNFα, related to Figure 3

(A) Schematic of knocking in a mCherry tag at the N-terminus of NEMO at the endogenous gene locus in U2OS cells using CRISPR.

(B) Immunoblotting of lysates from U2OS, U2OS$^{NEMO\ KO}$, and U2OS$^{mCherry\text{-}NEMO\ Knock\text{-}in}$ cells treated with IL-1β or TNFα. Cell lysates were analyzed by immunoblotting with the indicated antibodies. For immunoblotting of p-IκBα and IκBα, cells were stimulated with IL-1β or TNFα for 10 min and 20 min respectively.

A

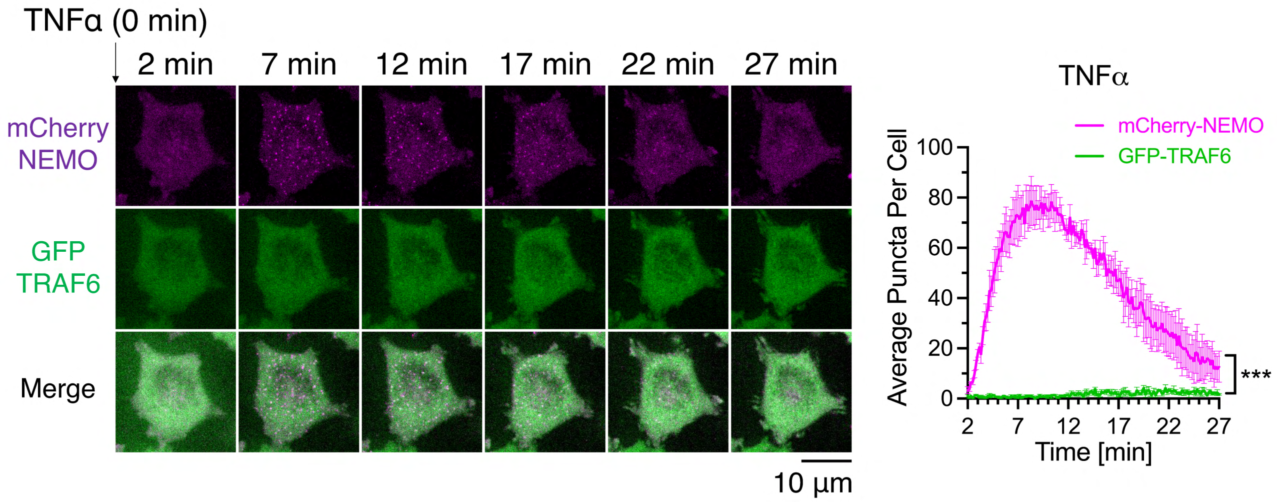

TNFα (0 min)
2 min
7 min
12 min
17 min
22 min
27 min
mCherry NEMO
GFP TRAF6
Merge
10 µm
TNFα
mCherry-NEMO
GFP-TRAF6
Average Puncta Per Cell
100
80
60
40
20
0
***
2
7
12
17
22
27
Time [min]

B

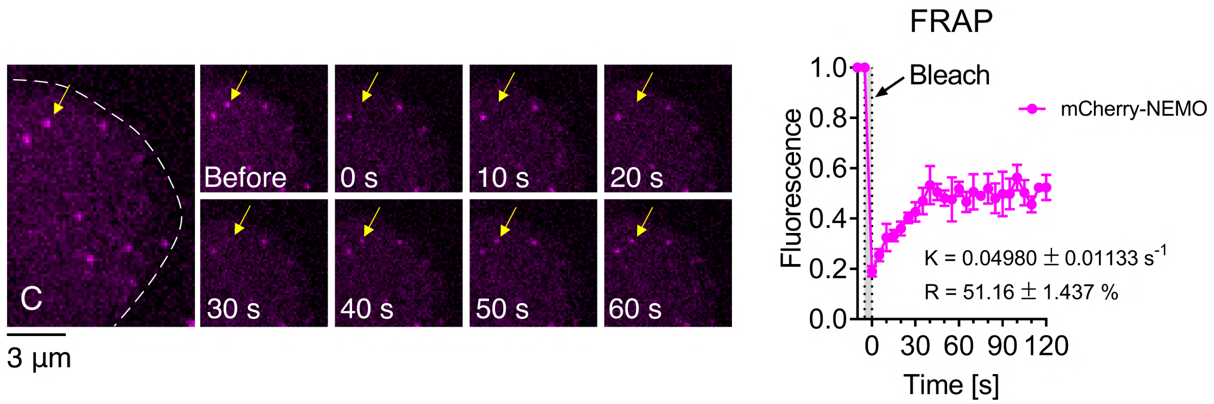

C
3 µm
Before
0 s
10 s
20 s
30 s
40 s
50 s
60 s
FRAP
Bleach
mCherry-NEMO
Fluorescence
1.0
0.8
0.6
0.4
0.2
0.0
K = 0.04980 ± 0.01133 s-1
R = 51.16 ± 1.437 %
0
30
60
90
120
Time [s]

Figure S5. NEMO formed liquid-like condensates in cells stimulated with TNFα, related to Figure 3

(A) Left panel: representative live cell images of NEMO condensates formation in HCT116$^{NEMO\ KO}$-mCherry-NEMO/GFP-TRAF6 cells stimulated by TNFα. Right panel: quantification of average numbers of condensates containing NEMO or TRAF6 per cell against time. Shown are means ± SEM. n = 3 cells. ***P < 0.0002, one-way analysis of variance (ANOVA). Results are from the same experiment as in Figure 6E-F.

(B) Left panel: representative micrographs of NEMO condensates before and after photobleaching (arrow, bleach site). NEMO condensates formed in HCT116$^{NEMO\ KO}$-mCherry-NEMO/GFP-TRAF6 cells stimulated by TNFα. Right panel: quantification of FRAP of NEMO puncta over a 120-s time course. K, exponential constant; R, normalized plateau after fluorescence recovery. C: cytoplasm. Shown are means ± SEM. n = 3 NEMO condensates.

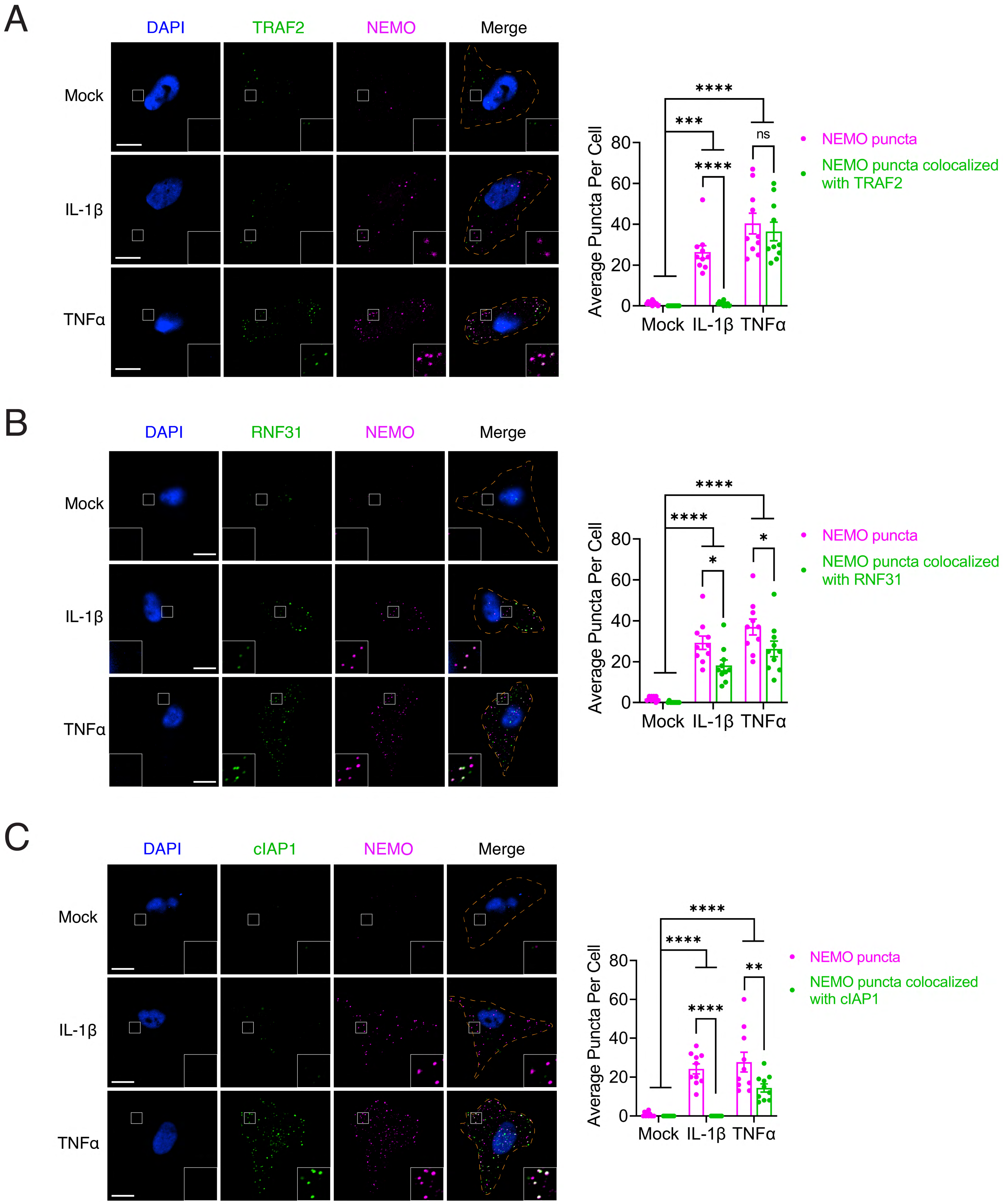
A
DAPI
TRAF2
NEMO
Merge
Mock
IL-1β
TNFα
Average Puncta Per Cell
0
20
40
60
80
****
***
****
ns
Mock
IL-1β
TNFα
NEMO puncta
NEMO puncta colocalized with TRAF2
B
DAPI
RNF31
NEMO
Merge
Mock
IL-1β
TNFα
Average Puncta Per Cell
****
****
*
*
NEMO puncta
NEMO puncta colocalized with RNF31
C
DAPI
cIAP1
NEMO
Merge
Mock
IL-1β
TNFα
Average Puncta Per Cell
****
****
****
**
NEMO puncta
NEMO puncta colocalized with cIAP1

Figure S6. NEMO condensates contain the E3 ligases TRAF2, RNF31 (HOIP), cIAP1 in cells stimulated with TNFα, related to Figure 3

(A) Left panel: immunofluorescence staining of TRAF2 in U2OS$^{mCherry\text{-}NEMO\ Knock\text{-}in}$ cells stimulated with either IL-1β or TNFα. Scale bars, 10 μm. Right panel: quantification of average numbers of NEMO puncta and those containing both NEMO and TRAF2 per cell.

(B) Left panel: immunofluorescence staining of endogenous NEMO and RNF31 (HOIP) in U2OS cells stimulated with either IL-1β or TNFα. Scale bars, 10 μm. Right panel: quantification of average numbers of NEMO puncta and those containing both NEMO and RNF31 (HOIP) per cell.

(C) Left panel: immunofluorescence staining of endogenous NEMO and cIAP1 in U2OS cells stimulated with either IL-1β or TNFα. Scale bars, 10 μm. Right panel: quantification of average numbers of NEMO puncta and those containing NEMO and cIAP1 per cell.

Data shown in (A), (B) and (C) are the means ± SEM. n = 10 cells. Two-way analysis of variance (ANOVA). n.s., $P > 0.0332$; *$P < 0.0332$; **$P < 0.0021$; ***, $P < 0.0002$; ****, $P < 0.0001$.

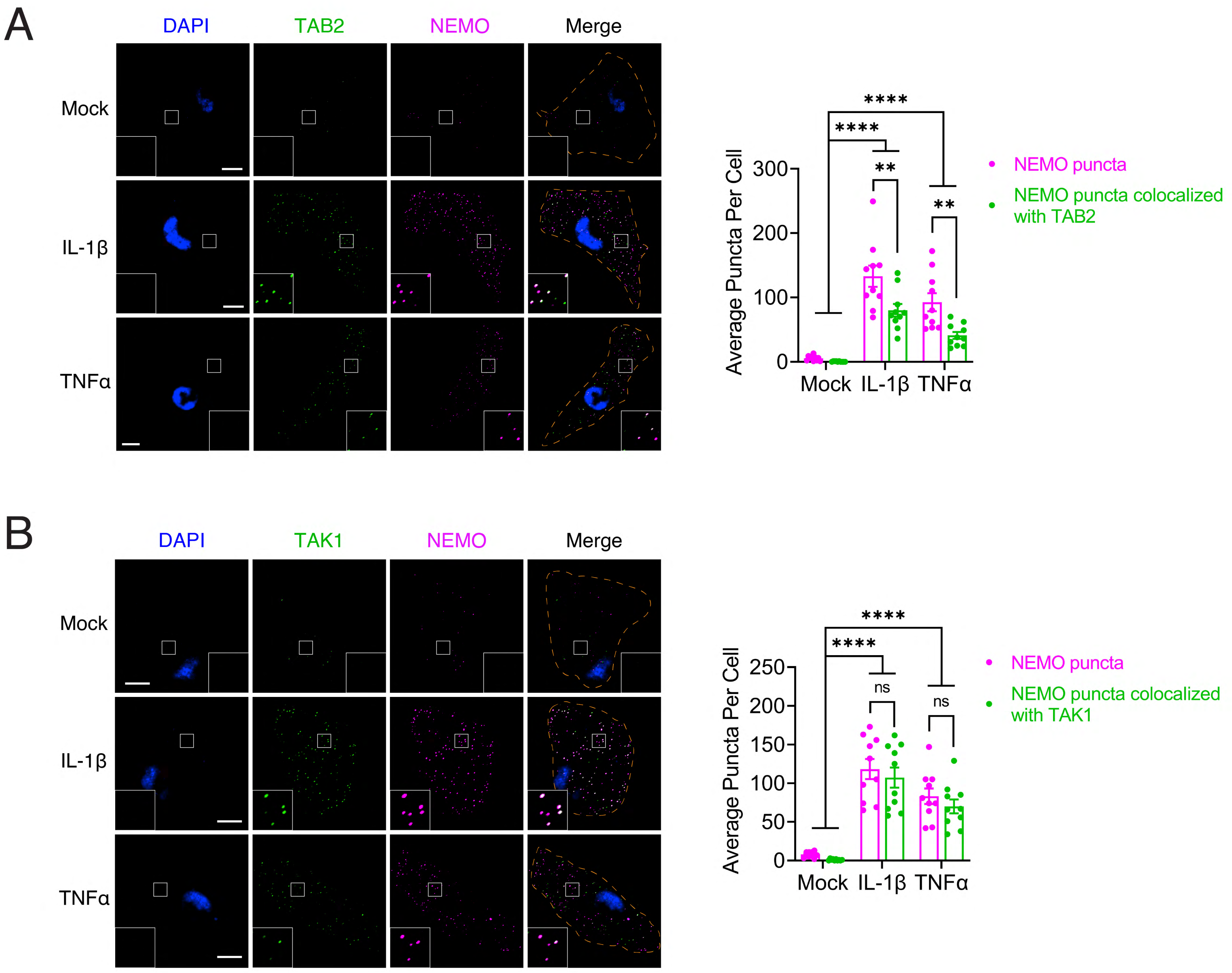
A
DAPI
TAB2
NEMO
Merge
Mock
IL-1β
TNFα
****
****
**
**
Average Puncta Per Cell
300
200
100
0
Mock
IL-1β
TNFα
NEMO puncta
NEMO puncta colocalized with TAB2
B
DAPI
TAK1
NEMO
Merge
Mock
IL-1β
TNFα
****
****
ns
ns
Average Puncta Per Cell
250
200
150
100
50
0
Mock
IL-1β
TNFα
NEMO puncta
NEMO puncta colocalized with TAK1

Figure S7. NEMO condensates contain TAB2 and TAK1 in cells stimulated with IL-1β or TNFα, related to Figure 4

(A) Left panel: immunofluorescence staining of endogenous NEMO and TAB2 in BJ-5ta cells stimulated with either IL-1β or TNFα. Scale bars, 10 μm. Right panel: quantification of average numbers of NEMO puncta and those containing both NEMO and TAB2 per cell.

(B) Left panel: immunofluorescence staining of endogenous NEMO and TAK1 in BJ-5ta cells stimulated with either IL-1β or TNFα. Scale bars, 10 μm. Right panel: quantification of average numbers of NEMO puncta and those containing both NEMO and TAK1 per cell.

Data shown in (A) and (B) are means ± SEM. n = 10 cells. Two-way analysis of variance (ANOVA). n.s., $P > 0.0332$; **$P < 0.0021$; ****, $P < 0.0001$.

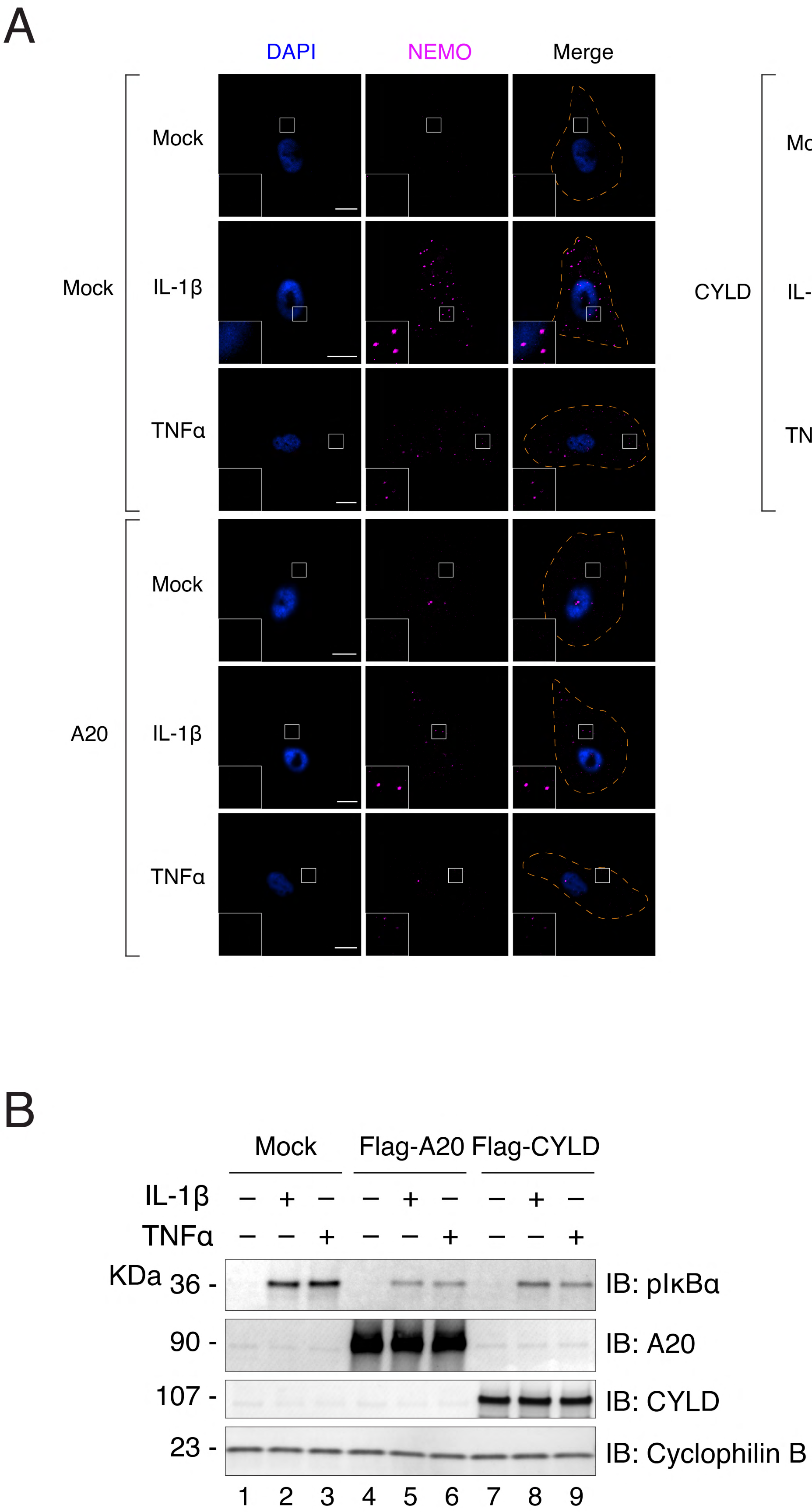
A
DAPI
NEMO
Merge
Mock
IL-1β
TNFα
Mock
A20
CYLD
B
Mock
Flag-A20
Flag-CYLD
IL-1β
TNFα
KDa
36 -
90 -
107 -
23 -
IB: pIκBα
IB: A20
IB: CYLD
IB: Cyclophilin B
1 2 3 4 5 6 7 8 9

Figure S8. Overexpression of A20 and CYLD reduces the numbers of NEMO condensates, related to Figure 4

(A) Fluorescent images of mCherry-NEMO condensates in U2OS$^{mCherry\text{-}NEMO\ Knock\text{-}in}$ cells stimulated with either IL-1β or TNFα. Prior to stimulation, cells were transfected with expression plasmids encoding Flag-A20 or Flag-CYLD as indicated. Scale bars, 10 μm.

(B) Immunoblotting of lysates from U2OS$^{mCherry\text{-}NEMO\ Knock\text{-}in}$ cells transiently overexpressing Flag-A20 or Flag-CYLD, which were stimulated with IL-1β or TNFα.

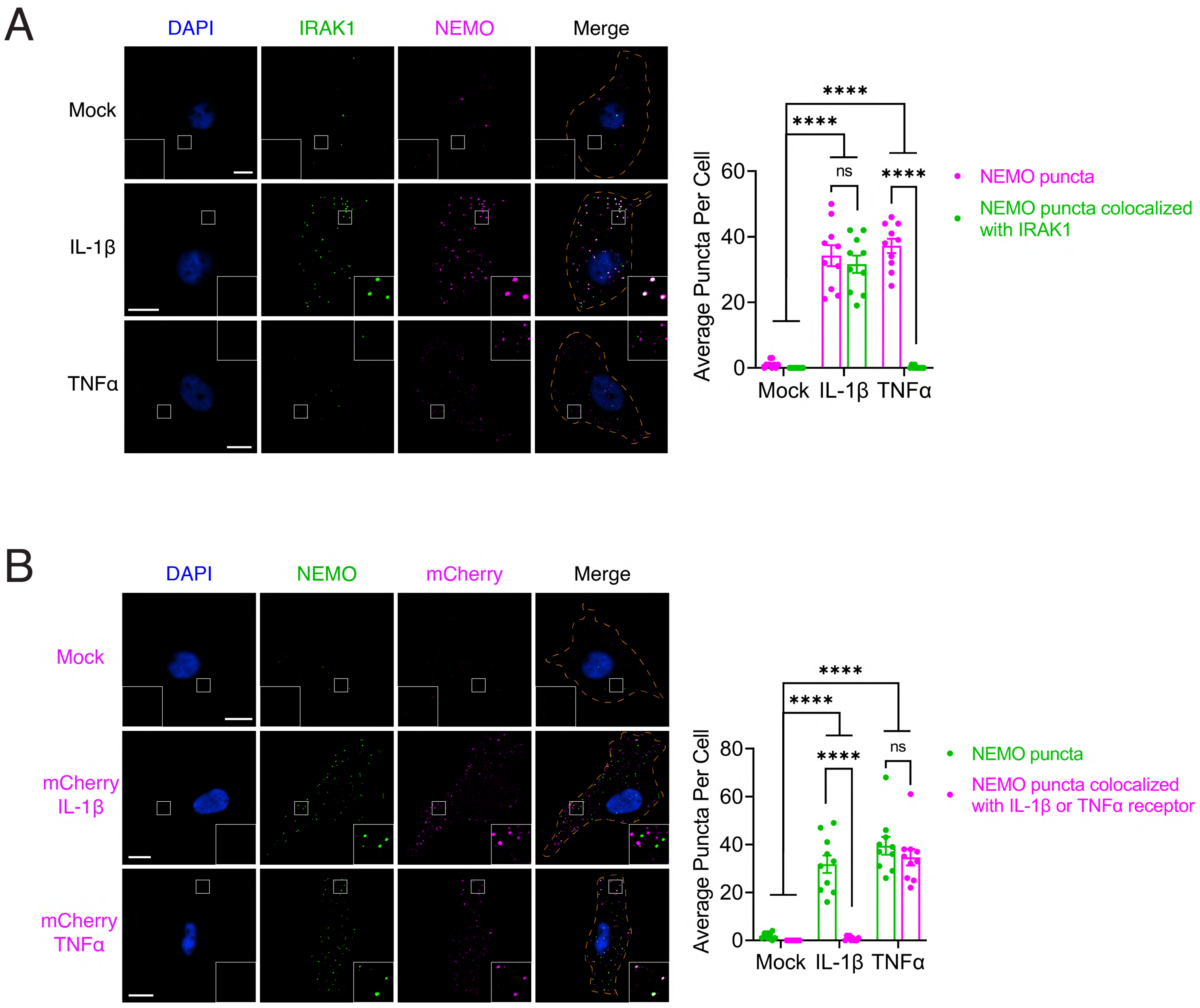
A
DAPI
IRAK1
NEMO
Merge
Mock
IL-1β
TNFα
Average Puncta Per Cell
0
20
40
60
Mock
IL-1β
TNFα
****
****
ns
****
NEMO puncta
NEMO puncta colocalized with IRAK1
B
DAPI
NEMO
mCherry
Merge
Mock
mCherry IL-1β
mCherry TNFα
Average Puncta Per Cell
0
20
40
60
80
Mock
IL-1β
TNFα
****
****
****
ns
NEMO puncta
NEMO puncta colocalized with IL-1β or TNFα receptor

Figure S9. NEMO condensates co-localized with TNFα receptors but not IL-1β receptor in response to stimulation, related to Figure 4

(A) Left panel: immunofluorescence staining of IRAK1 in U2OS$^{mCherry\text{-}NEMO\ knock\text{-}in}$ cells stimulated with either IL-1β or TNFα. Scale bars, 10 μm. Right panel: quantification of average numbers of NEMO puncta and those containing both NEMO and IRAK1 per cell.

(B) Left panel: immunofluorescence staining of endogenous NEMO in U2OS cells stimulated with either mCherry-IL-1β or mCherry-TNFα. Scale bars, 10 μm. Right panel: quantification of average numbers of NEMO puncta and those containing both NEMO and mCherry-IL-1β or mCherry-TNFα per cell.

Data shown in (A) and (B) are the means ± SEM. n = 10 cells. Two-way analysis of variance (ANOVA). n.s., $P > 0.0332$; ****, $P < 0.0001$.

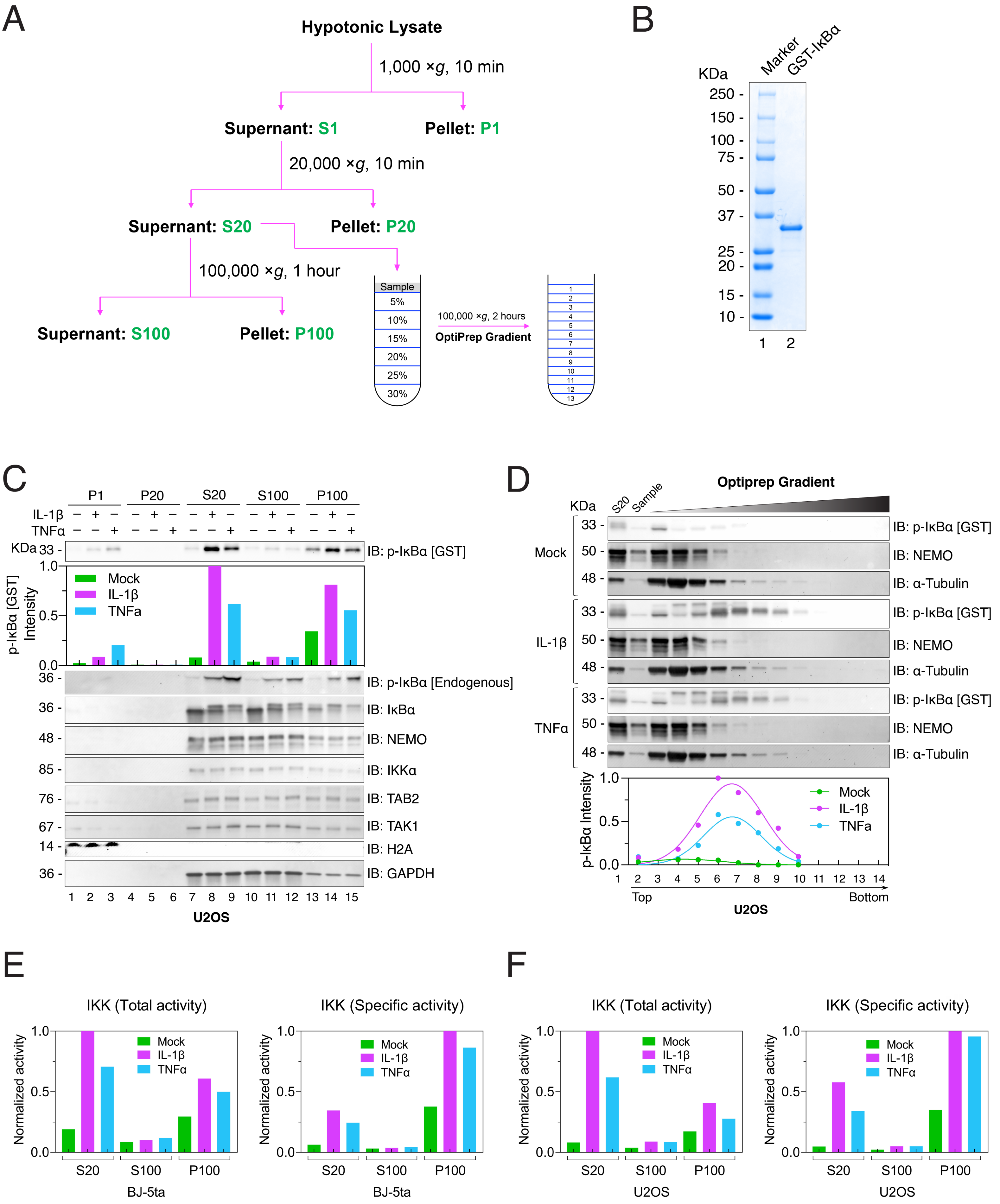
A
Hypotonic Lysate
1,000 ×g, 10 min
Supernant: S1
Pellet: P1
20,000 ×g, 10 min
Supernant: S20
Pellet: P20
100,000 ×g, 1 hour
Supernant: S100
Pellet: P100
Sample
5%
10%
15%
20%
25%
30%
100,000 ×g, 2 hours
OptiPrep Gradient
1
2
3
4
5
6
7
8
9
10
11
12
13
B
KDa
Marker
GST-IκBα
250 -
150 -
100 -
75 -
50 -
37 -
25 -
20 -
15 -
10 -
1
2
C
P1
P20
S20
S100
P100
IL-1β
TNFα
KDa
33 -
IB: p-IκBα [GST]
p-IκBα [GST] Intensity
1.0
0.5
0.0
Mock
IL-1β
TNFa
36 -
IB: p-IκBα [Endogenous]
36 -
IB: IκBα
48 -
IB: NEMO
85 -
IB: IKKα
76 -
IB: TAB2
67 -
IB: TAK1
14 -
IB: H2A
36 -
IB: GAPDH
1 2 3 4 5 6 7 8 9 10 11 12 13 14 15
U2OS
D
Optiprep Gradient
KDa
S20
Sample
Mock
IL-1β
TNFα
33 -
50 -
48 -
IB: p-IκBα [GST]
IB: NEMO
IB: α-Tubulin
p-IκBα Intensity
1.0
0.5
0.0
Mock
IL-1β
TNFa
1 2 3 4 5 6 7 8 9 10 11 12 13 14
Top
Bottom
U2OS
E
IKK (Total activity)
IKK (Specific activity)
Normalized activity
1.0
0.5
0.0
Mock
IL-1β
TNFα
S20
S100
P100
BJ-5ta
F
IKK (Total activity)
IKK (Specific activity)
Normalized activity
Mock
IL-1β
TNFα
S20
S100
P100
U2OS

Figure S10. IKK activities were enriched in subcellular fractions containing NEMO condensates after cells were stimulated with IL-1β or TNFα, related to Figure 4

(A) Schematic of subcellular fractionation procedures.

(B) Coomassie blue staining of purified recombinant GST-IκBα (amino acids 1-62, 33 KDa).

(C) Subcellular fractionation of IKK activity in cells stimulated with IL-1β or TNFα. U2OS cells stimulated with IL-1β or TNFα were fractionated by differential centrifugation as depicted in (A). Fractions were incubated with recombinant GST-IκBα in a kinase reaction buffer, followed by immunoblotting with an antibody against p-IκBα. Bar plot represents the quantification of p-GST-IκBα. Fractions were also analyzed by immunoblotting (IB) with the indicated antibodies.

(D) The S20 fractions from (C) were further separated by OptiPrep gradient ultracentrifugation, and IKK activity in different fractions was measured as in (C). The 36 kDa upper band detected by p-IκBα is endogenous p-IκBα, whereas the lower band is p-GST-IκBα, which reports the IKK activity in the in vitro kinase assay. Plot shows quantification of IKK activity after OptiPrep gradient ultracentrifugation. Curves represent dots fitting by a gaussian distribution model (Least square fit).

(E) Left panel: quantification of IKK total activity in subcellular fractions as shown in Figure 4F. The total volume of P100 was half of S100 volume. Right panel: quantification of IKK specific activity (dividing level of p-GST-IκBα by that of NEMO) in subcellular fractions as shown in Figure 4F.

(F) Quantification of IKK total activity and specific activity was similar to (E) except that subcellular fractions were from U2OS cells as shown in panel (C) of this figure.

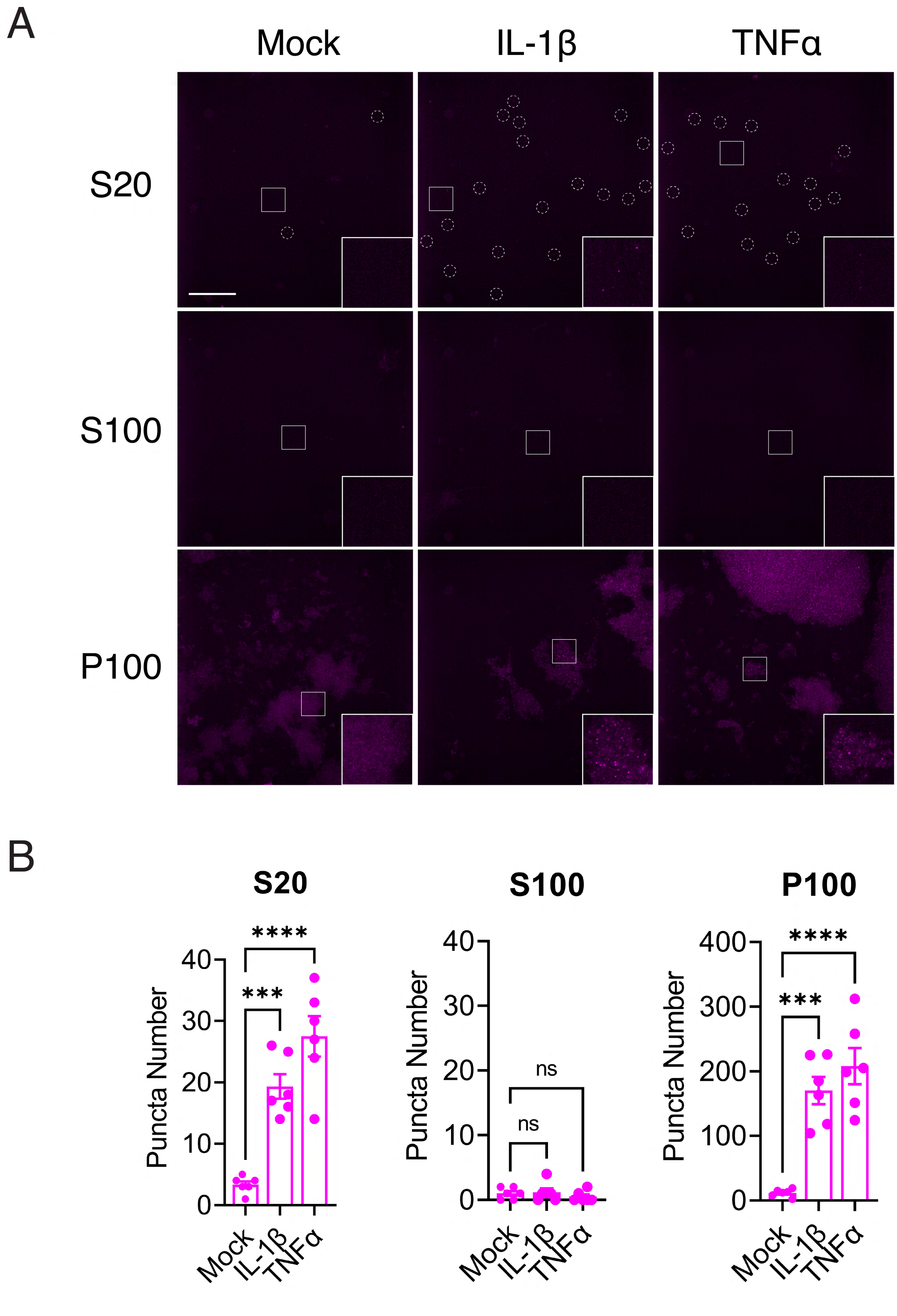

A
Mock
IL-1β
TNFα
S20
S100
P100
B
S20
S100
P100
Puncta Number
***
****
ns
ns
Mock
IL-1β
TNFα
0
10
20
30
40
100
200
300
400

Figure S11. NEMO condensates were enriched in the pellet (P100) after centrifugation, related to Figure 4

(A) Representative fluorescent images of NEMO condensates in each subcellular fraction. U2OS$^{\text{mCherry-NEMO knock-in}}$ cells stimulated with IL-1β or TNFα were fractionated by differential centrifugation as depicted in Figure S10A. mCherry-NEMO condensates in each fraction were examined under a confocal fluorescent microscope.

(B) Quantification of mCherry-NEMO condensates in each fraction shown in (A). Scale bars, 50 μm. Data are means ± SEM. n = 6 areas. One-way analysis of variance (ANOVA). n.s., $P > 0.0332$; ***, $P < 0.0002$; ****, $P < 0.0001$.

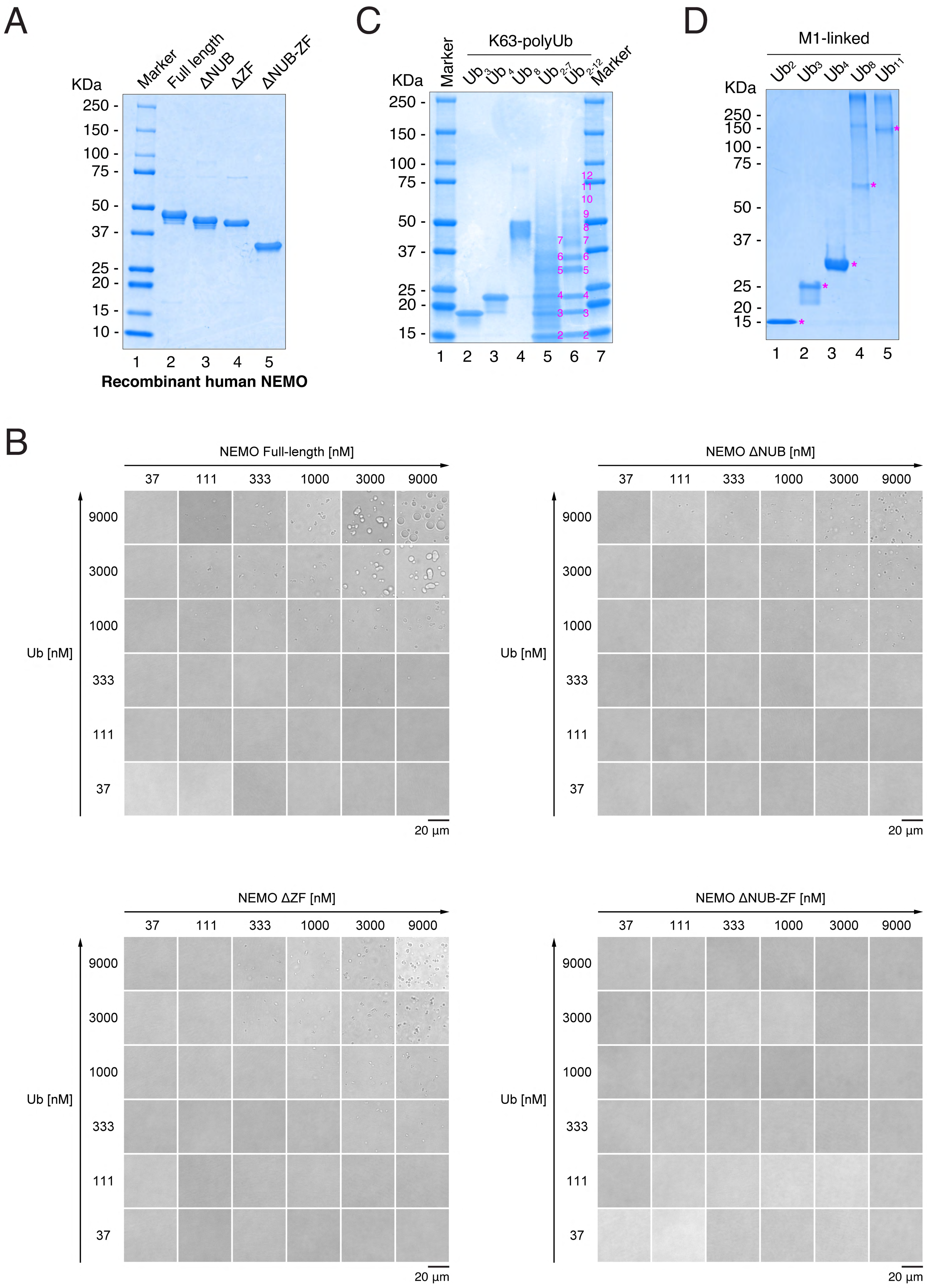

A
KDa
Marker
Full length
ΔNUB
ΔZF
ΔNUB-ZF
250
150
100
75
50
37
25
20
15
10
1 2 3 4 5
Recombinant human NEMO
C
K63-polyUb
Marker
Ub3
Ub4
Ub8
Ub2-7
Ub2-12
Marker
1 2 3 4 5 6 7
D
M1-linked
Ub2
Ub3
Ub4
Ub8
Ub11
1 2 3 4 5
B
NEMO Full-length [nM]
NEMO ΔNUB [nM]
NEMO ΔZF [nM]
NEMO ΔNUB-ZF [nM]
37 111 333 1000 3000 9000
Ub [nM]
20 µm

Figure S12. Multivalent interactions between NEMO and polyUb drive their phase separation, related to Figure 5

(A) Coomassie blue staining of purified full-length human NEMO (48 KDa), ΔNUB (45 KDa), ΔZF (45 KDa), and ΔNUB-ZF (34 KDa).

(B) Representative bright field images of NEMO liquid droplets induced by K63-polyUb chains. Liquid droplets were imaged after mixing of human FL-NEMO or its mutants with polyubiquitin chains synthesized in a reaction mixture containing E1, E2 (UBC13/UEV1A), E3 (TRAF6) and the indicated concentrations of ubiquitin. These images correspond to the phase separation diagram in Figure 5C.

(C) Coomassie blue staining of K63-linked Ub_3, Ub_4, Ub_8, $Ub_{2\text{-}7}$ and $Ub_{2\text{-}12}$. Numbers (magenta color) indicate the bands of Ub_2 to Ub_7 or Ub_2 to Ub_{12}. K63-linked $Ub_{2\text{-}7}$ appeared to have some minor smears that may represent impurity.

(D) Coomassie blue staining of linear Ub_2, Ub_3, Ub_4, Ub_8, and Ub_{11}. Asterisks (magenta color) indicate the proteins of interest.

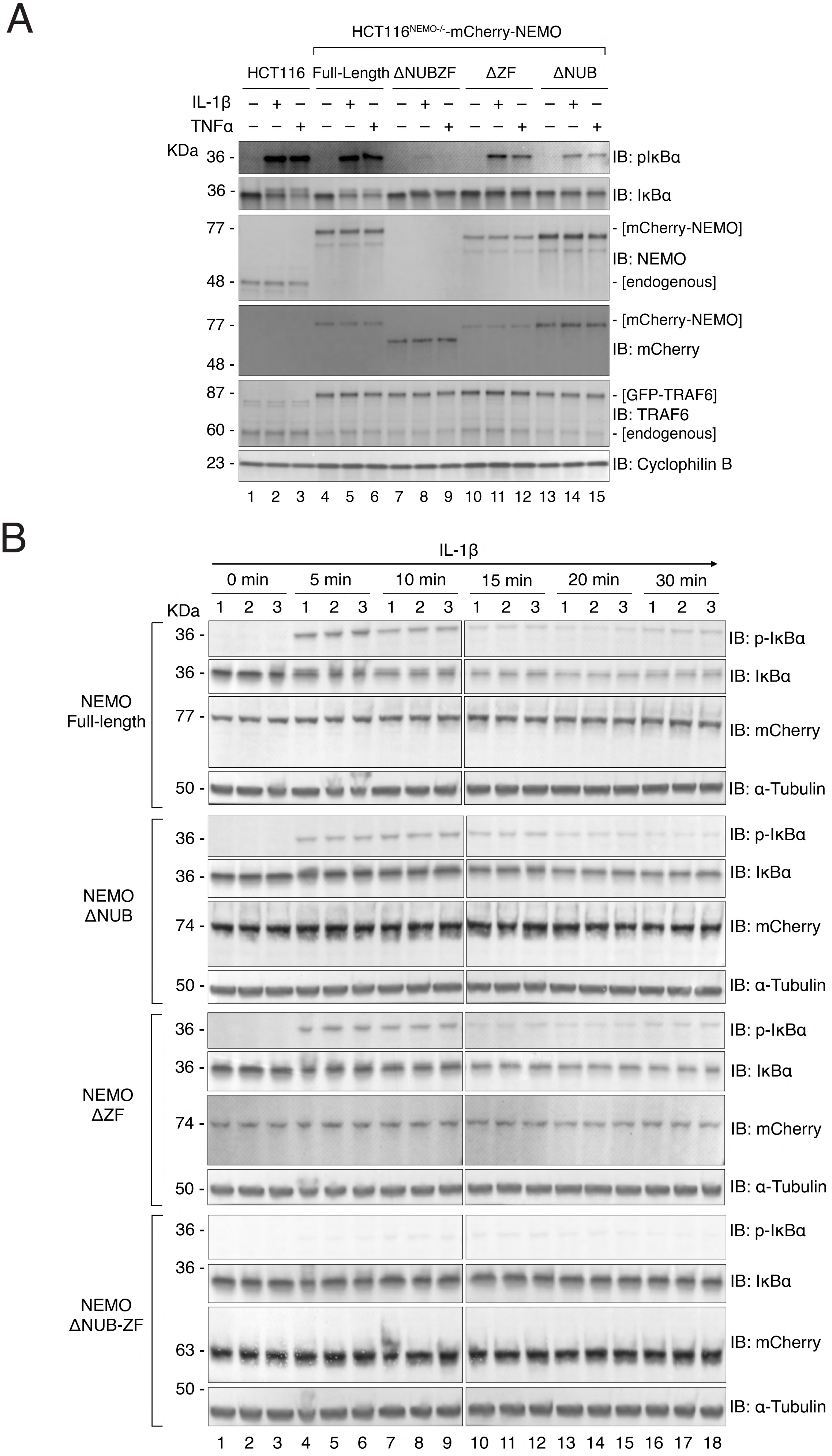
A
HCT116NEMO-/--mCherry-NEMO
HCT116
Full-Length
ΔNUBZF
ΔZF
ΔNUB
IL-1β
TNFα
KDa
IB: pIκBα
IB: IκBα
[mCherry-NEMO]
IB: NEMO
[endogenous]
IB: mCherry
[GFP-TRAF6]
IB: TRAF6
IB: Cyclophilin B
B
IL-1β
0 min
5 min
10 min
15 min
20 min
30 min
NEMO Full-length
NEMO ΔNUB
NEMO ΔZF
NEMO ΔNUB-ZF
IB: p-IκBα
IB: α-Tubulin

Figure S13. Multivalent interactions between NEMO and polyUb lead to NEMO phase separation and IKK activation in cells, related to Figure 6

(A) Immunoblotting of lysates from HCT116$^{NEMO\ KO}$-GFP-TRAF6 cells reconstituted with mCherry-tagged human NEMO or indicated mutants. Cells were stimulated with IL-1β or TNFα for 10 min.

(B) Similar to (A) except that cells were stimulated with IL-1β for indicated time, with each time point analyzed in triplicate. Data were used to plot the graphs shown in Figure 6C-D.

A

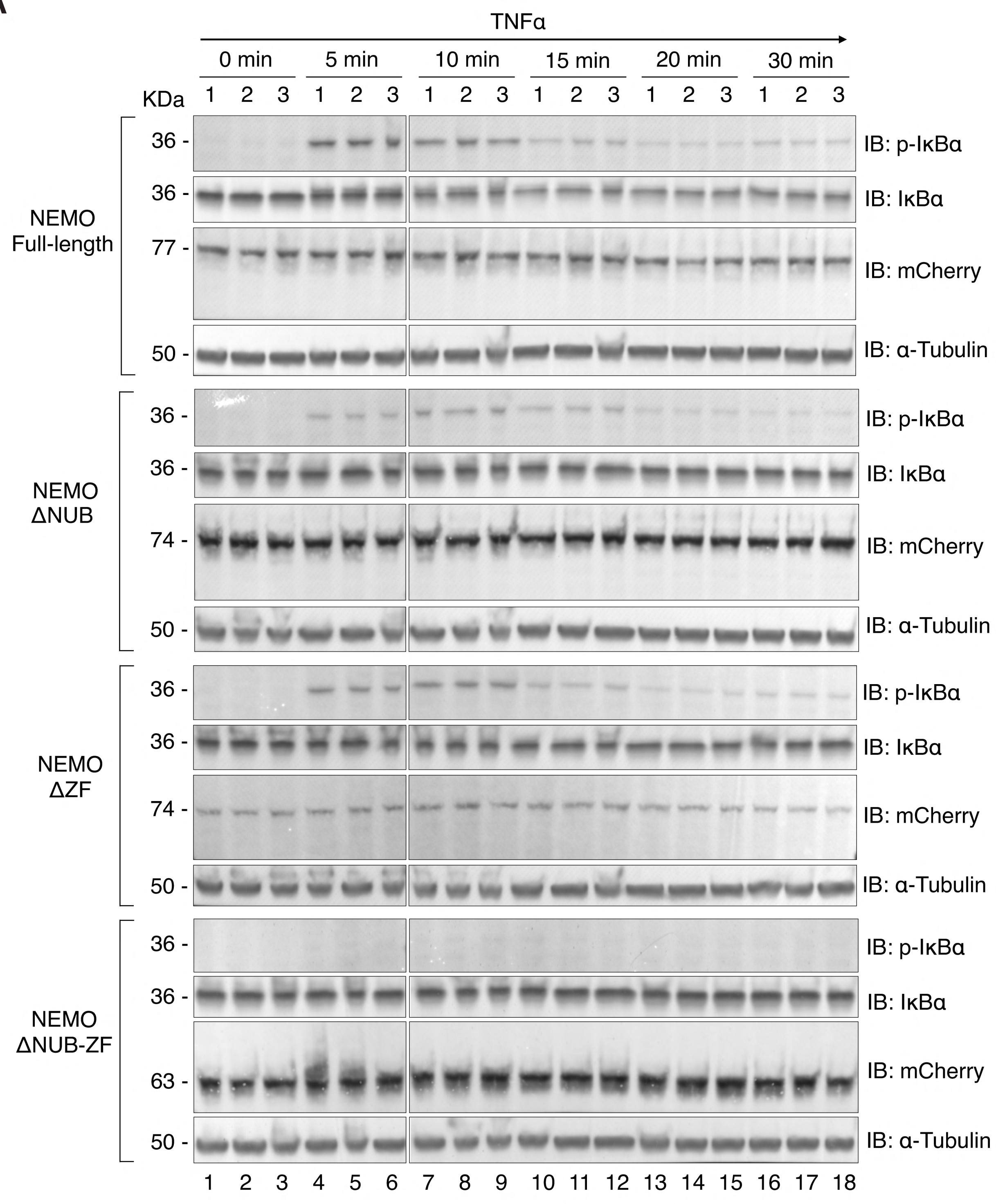
TNFα
0 min
5 min
10 min
15 min
20 min
30 min
1 2 3 1 2 3 1 2 3 1 2 3 1 2 3 1 2 3
KDa
NEMO Full-length
36 -
36 -
77 -
50 -
NEMO ΔNUB
36 -
36 -
74 -
50 -
NEMO ΔZF
36 -
36 -
74 -
50 -
NEMO ΔNUB-ZF
36 -
36 -
63 -
50 -
IB: p-IκBα
IB: IκBα
IB: mCherry
IB: α-Tubulin
1 2 3 4 5 6 7 8 9 10 11 12 13 14 15 16 17 18

Figure S14. Multivalent interactions between NEMO and polyUb led to NEMO phase separation and IKK activation in cells stimulated with TNFα, related to Figure 6

Experiments and cell lines were similar to Figure S13B except that cells were stimulated with TNFα for indicated time. Data were used to plot the graphs shown in Figure 6G-H.

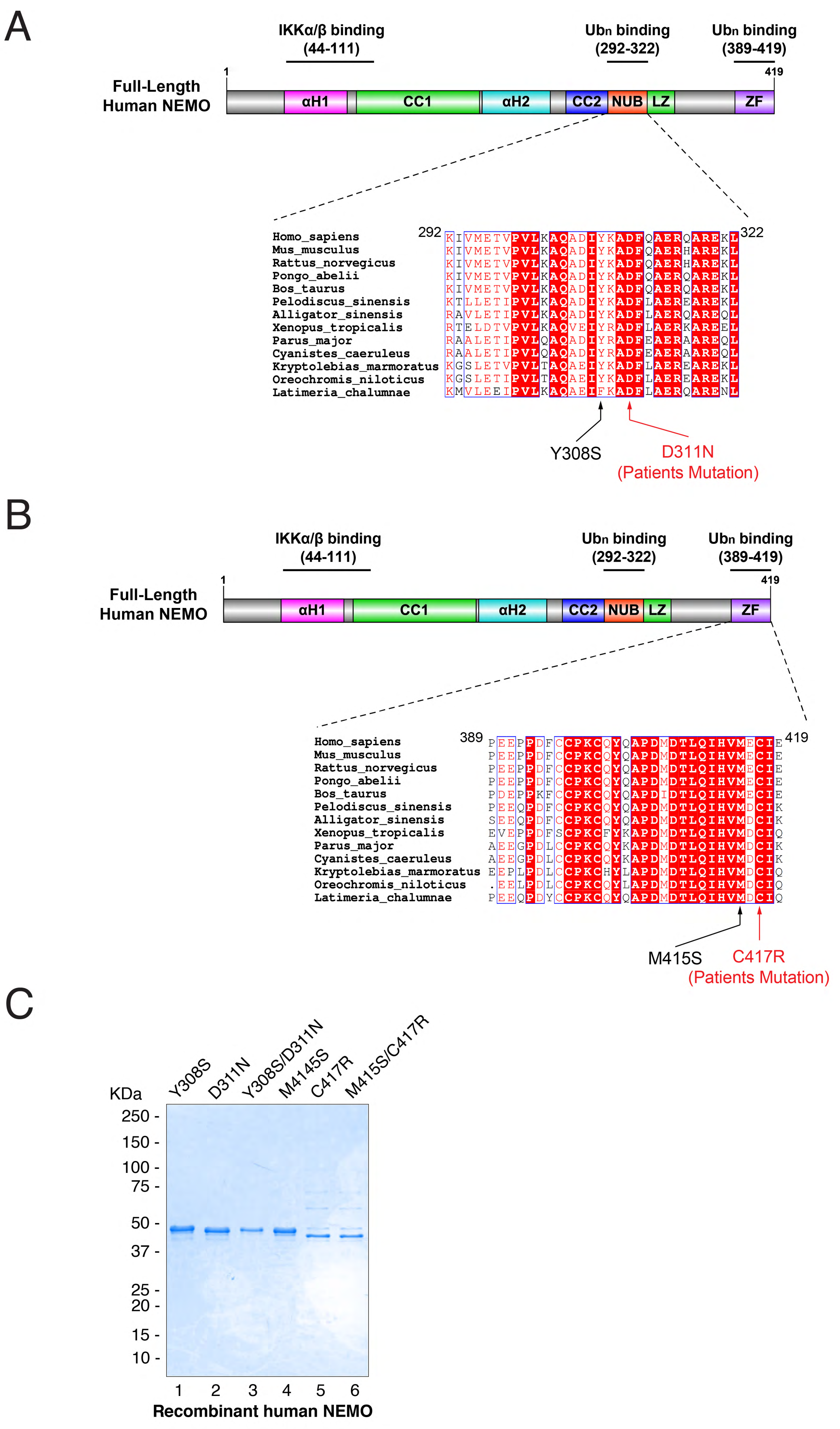
A
IKKα/β binding (44-111)
Ubn binding (292-322)
Ubn binding (389-419)
Full-Length Human NEMO
1
419
αH1
CC1
αH2
CC2
NUB
LZ
ZF
Homo_sapiens
Mus_musculus
Rattus_norvegicus
Pongo_abelii
Bos_taurus
Pelodiscus_sinensis
Alligator_sinensis
Xenopus_tropicalis
Parus_major
Cyanistes_caeruleus
Kryptolebias_marmoratus
Oreochromis_niloticus
Latimeria_chalumnae
292
322
Y308S
D311N (Patients Mutation)
B
IKKα/β binding (44-111)
Ubn binding (292-322)
Ubn binding (389-419)
Full-Length Human NEMO
1
419
αH1
CC1
αH2
CC2
NUB
LZ
ZF
Homo_sapiens
Mus_musculus
Rattus_norvegicus
Pongo_abelii
Bos_taurus
Pelodiscus_sinensis
Alligator_sinensis
Xenopus_tropicalis
Parus_major
Cyanistes_caeruleus
Kryptolebias_marmoratus
Oreochromis_niloticus
Latimeria_chalumnae
389
419
M415S
C417R (Patients Mutation)
C
KDa
Y308S
D311N
Y308S/D311N
M4145S
C417R
M415S/C417R
250 -
150 -
100 -
75 -
50 -
37 -
25 -
20 -
15 -
10 -
1 2 3 4 5 6
Recombinant human NEMO

Figure S15. NEMO ubiquitin-binding mutants, related to Figure 7

(A) Sequence alignment of NEMO ubiquitin binding (NUB) domain across multiple species. The mutations Y308S and D311N impair the binding of NEMO to polyUb.

(B) Sequence alignment of NEMO zinc finger (ZF) domain across multiple species. The mutations M415S and C417R impair the binding of NEMO to polyUb.

(C) Coomassie blue staining of purified recombinant human full-length NEMO protein bearing single or double mutations as indicated.

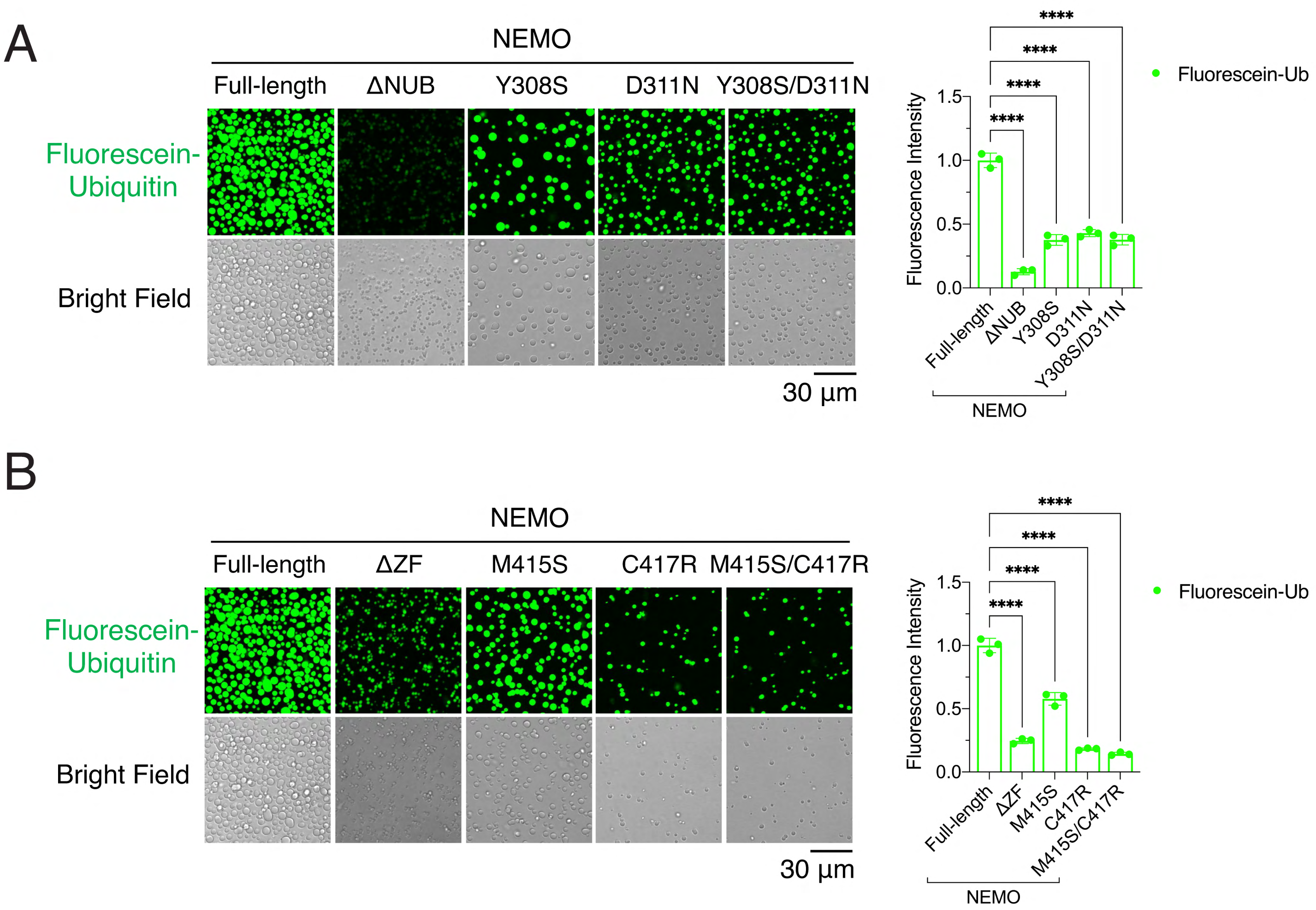
A
NEMO
Full-length
ΔNUB
Y308S
D311N
Y308S/D311N
Fluorescein-Ubiquitin
Bright Field
30 µm
Fluorescence Intensity
1.5
1.0
0.5
0.0
****
****
****
****
Fluorescein-Ub
Full-length
ΔNUB
Y308S
D311N
Y308S/D311N
NEMO
B
NEMO
Full-length
ΔZF
M415S
C417R
M415S/C417R
Fluorescein-Ubiquitin
Bright Field
30 µm
Fluorescence Intensity
1.5
1.0
0.5
0.0
****
****
****
****
Fluorescein-Ub
Full-length
ΔZF
M415S
C417R
M415S/C417R
NEMO

Figure S16. NEMO mutants linked to human diseases are defective in phase separation with polyubiquitin chains, related to Figure 7

(A) and (B) Left panel: representative images of phase separation after incubating NEMO or its mutants with K63-polyUb synthesized in a reaction containing E1, UBC13/UEV1A, TRAF6, ubiquitin and fluorescein ubiquitin. Right panel: quantification of fluorescence intensity of liquid droplets. Shown are means ± SD. n = 3 areas. One-way analysis of variance (ANOVA); ****, $P < 0.0001$. These images are from the same experiment as in Figure 5B.
